# Current Advances in Lipid and Polymeric Antimicrobial Peptide Delivery Systems and Coatings for the Prevention and Treatment of Bacterial Infections

**DOI:** 10.3390/pharmaceutics13111840

**Published:** 2021-11-02

**Authors:** Miriam E. van Gent, Muhanad Ali, Peter H. Nibbering, Sylvia N. Kłodzińska

**Affiliations:** 1 Department of Infectious Diseases, Leiden University Medical Center, 2300 RC Leiden, The Netherlands; M.E.van_Gent@lumc.nl (M.E.v.G.); M.Ali2@lumc.nl (M.A.); P.H.Nibbering@lumc.nl (P.H.N.); 2 Center for Biopharmaceuticals and Biobarriers in Drug Delivery, Department of Pharmacy, Faculty of Health and Medical Sciences, University of Copenhagen, Universitetsparken 2, DK-2100 Copenhagen, Denmark

**Keywords:** antimicrobial peptide, drug delivery system, liposome, PLGA nanoparticle, nanogel, antimicrobial coating, release profile, bacteria, biofilm

## Abstract

Bacterial infections constitute a threat to public health as antibiotics are becoming less effective due to the emergence of antimicrobial resistant strains and biofilm and persister formation. Antimicrobial peptides (AMPs) are considered excellent alternatives to antibiotics; however, they suffer from limitations related to their peptidic nature and possible toxicity. The present review critically evaluates the chemical characteristics and antibacterial effects of lipid and polymeric AMP delivery systems and coatings that offer the promise of enhancing the efficacy of AMPs, reducing their limitations and prolonging their half-life. Unfortunately, the antibacterial activities of these systems and coatings have mainly been evaluated in vitro against planktonic bacteria in less biologically relevant conditions, with only some studies focusing on the antibiofilm activities of the formulated AMPs and on the antibacterial effects in animal models. Further improvements of lipid and polymeric AMP delivery systems and coatings may involve the functionalization of these systems to better target the infections and an analysis of the antibacterial activities in biologically relevant environments. Based on the available data we proposed which polymeric AMP delivery system or coatings could be profitable for the treatment of the different hard-to-treat infections, such as bloodstream infections and catheter- or implant-related infections.

## 1. Introduction

Antibiotics are highly successful drugs that save the lives of millions of people yearly and are essential in many important medical procedures, such as transplantation, novel tumor treatments and complex surgical procedures, including implantation of artificial body parts. Unfortunately, a growing number of infections are hard-to-treat with current antibiotics, due to the emergence of antimicrobial resistant (AMR) strains [1] and/or biofilm and persister formation. Biofilms are communities of bacteria (and other micro-organisms) that are protected from the actions of hostile factors, including antibiotics, by a self-produced extracellular matrix comprised of polysaccharides, proteins and DNA [2]. In addition, bacteria hiding in deeper layers of the biofilm may transfer to a metabolically inactive state, so-called persisters, which are tolerant to the actions of many antibiotics [3]. These persisters cannot be treated by antibiotics that target routes involved in the metabolism of bacteria, but only by high concentrations of antibiotics that target essential features of the bacteria, such as their membrane. Moreover, bacteria are able to hide inside host cells [4] to avoid the action of antibiotics as well as factors of the immune systems. Based on these considerations, there is an urgent need for novel agents that are effective against a broad range of AMR pathogenic bacteria, residing in a biofilm or host cells.

**Figure 1:**
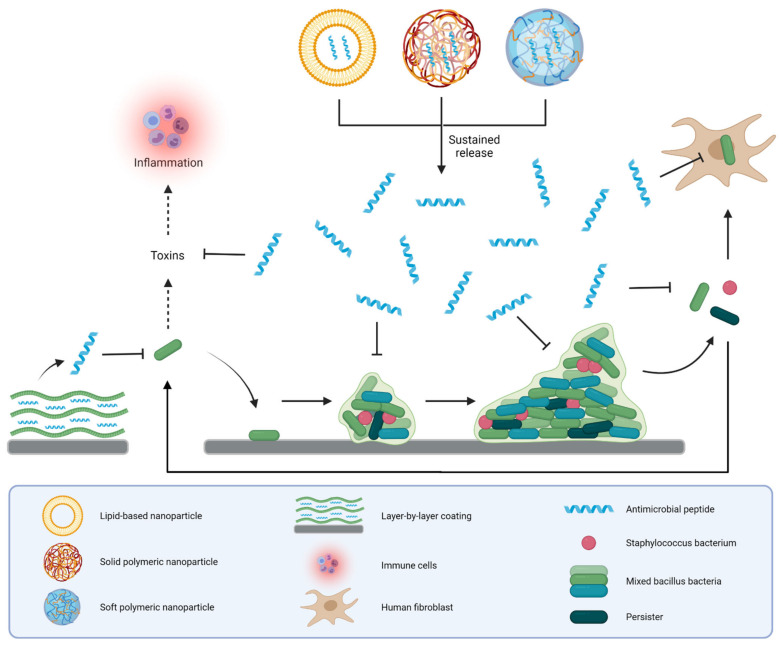
Simplified schematic representation of the antibacterial effects of antimicrobial peptides released from lipid and polymeric drug delivery systems and coatings.

Antimicrobial peptides (AMPs) are promising candidates for the development of novel therapies to combat hard-to-treat infections [5,6]. AMPs are part of the defense against infectious agents in a wide range of organisms, including humans; they contain 10 to 60 amino acids and are often positively charged. Based on their structure, AMPs can be divided into four general classes: linear α-helical peptides, β-sheet peptides, linear extension structured peptides and peptides containing both α-helices and β-sheets [7]. AMPs have a mode of action different from current antibiotics as they kill a broad range of bacteria by interacting with and subsequently destabilizing the plasma membrane of AMR bacteria, including persisters [8,9]. In addition, multiple AMPs have shown to be effective against bacteria residing in biofilms [9,10], although penetration of AMPs into the biofilm can be hampered by electrostatic interactions between cationic residues of the peptide and anionic components, like extracellular polysaccharides and DNA, produced by the biofilm-residing bacteria [11]. Furthermore, several AMPs have been shown to neutralize bacterial toxins, including bacterial cell wall components that trigger an inflammatory response [12]. Importantly, the risk of resistance development to AMPs is considered low due to their nonspecific mode of action [13]. In addition to these antibacterial actions, AMPs may contribute to the defense against infections by their ability to attract phagocytes and T cells to the site of infection, to activate immune cells, and to modulate macrophage and dendritic cell differentiation and activation [14]. A schematic representation of the interactions between AMPs and bacteria as well as cells of the immune system is provided in Figure 1.

Unfortunately, the use of AMPs in vivo is limited by several factors connected to their peptidic nature, including a low selectivity towards bacterial cells over mammalian cells, rapid removal from the circulation, low physical stability in body fluids and at infection sites due to the degradation by enzymes and stomach acids, limited bioavailability through binding with (plasma) proteins and other molecules, and low tissue penetration [15,16,17]. One approach to improve the therapeutic potential of AMPs is to encapsulate them into a drug delivery system (DDS). These DDSs can circumvent several limitations associated with AMPs. First, DDSs that encapsulate AMPs within their core, shield the peptide from its environment, thereby preventing premature degradation of AMPs by extracellular components (for instance hydrolytic enzymes) and limiting binding of AMPs to components, like proteins, in the extracellular milieu [18]. Second, some DDSs release the AMP in a sustained manner, which allows for the control of AMP concentration levels in the therapeutic range and reduces the toxicity associated with the AMP [19]. Third, DDSs can assist AMP transport across cellular barriers like mucosae and skin [20]. Fourth, DDSs can improve biofilm penetration, intracellular retention and the subcellular distribution of AMPs either by themselves or by offering the possibility for surface functionalization, such as conjugation with targeting ligands, e.g., polyethylene glycol or cell penetrating peptides and biofilm-targeting compounds, such as N-acetyl-cysteine [20,21,22,23]. Last, DDSs provide the possibility to tune several properties of the delivery system, like peptide release profile, size and surface charge by altering essential formulation parameters, such as organic solvents, polymer length and surfactants. All of these features underscore the potential of DDSs for AMP delivery in the clinic. In the case of medical devices, such as implants and catheters, AMPs could be incorporated in coatings. Several AMP coatings have been developed to protect medical devices from colonization by biofilm-forming bacteria, thus preventing associated infections [24]. In order to prevent or combat pathogens infecting tissues surrounding medical devices, coatings that release AMPs in a controlled fashion over a long time span have been developed [25,26].

In this review, we will discuss the main chemical characteristics, the release profile and, most importantly, the antibacterial and antibiofilm activities as well as toxicity of the lipid and polymeric AMP delivery systems and coatings reported in the literature. In addition, we attempt to identify which delivery system and/or coating is profitable for AMP delivery in the fight against and/or prevention of the major hard-to-treat infections, including bloodstream and deep-seated infections, catheter-related and implant-associated infections, pulmonary tract infections, and complex wound infections (such as diabetic ulcers and burn wounds).

## 2. Literature Strategy

This review is based on studies in English without restrictions regarding year of publication; however, we mainly focused on the recent literature spanning from 2016 to 2021. References were sourced from electronic databases PubMed, Embase, Web of Science and Cochrane Library. A two-stranded search strategy was applied, where the main search terms included “antimicrobial peptides”, “type of nano formulations”, “drug delivery system”, “coatings” and “infection”. Subsequently, titles and abstracts were screened to obtain the relevant literature.

## 3. Nanoparticles

Drug delivery technology has advanced significantly since the introduction of the first controlled-release formulation in the 1950’s [27], and is utilized for the formulation of AMPs more and more frequently, with an array of DDSs developed and tested for improving the bioavailability of AMPs and delivery to bacterial infections. Although a range of drug delivery approaches allow for a controlled release and targeted delivery, nanosized particle systems, such as liposomes, lipid-based nanostructures, polymeric nanoparticles and nanogels are particularly interesting as they allow a higher biofilm penetration than macro-sized formulations [28]. Various fabrication methods for these nanoparticles have been developed to match the used material to properties of the encapsulated cargo and its intended target. Extensive reviews summarizing these methods are available in the literature [29,30,31,32,33]. Nanoparticles, whether lipid or polymeric, typically show a particle size range of 1 to 100 nm [34], though some use the term for larger particles, up to 500 nm. However, as the efficiency and usefulness of drug delivery systems are not based only on their particle size [35], in this review nanoparticles are defined as particles in the range of 1–500 nm, with some relevant reports on microparticles also included.

Provided below is an overview of lipid and polymeric nanoparticle delivery systems encapsulating AMPs and coatings developed in the last 5 years together with a discussion regarding some of the advantages and limitations of these systems against in vitro and in vivo infections.

### 3.1. Lipid-Based Nanoparticles

#### 3.1.1. Liposomes

**Figure 2:**
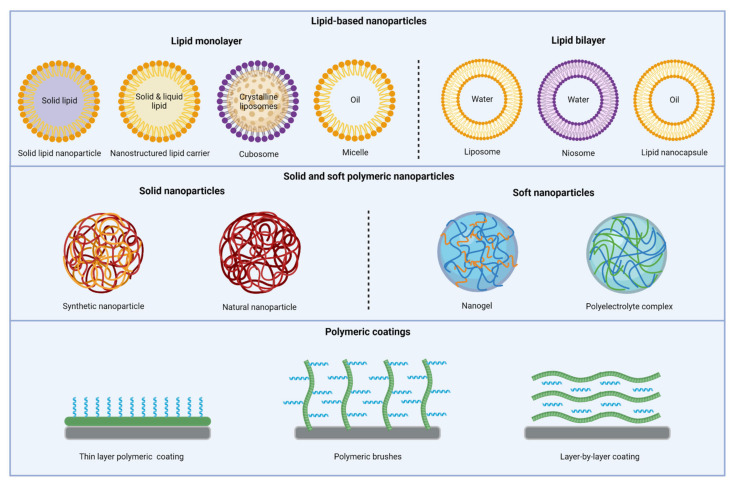
Schematic overview of the structures of various types of lipid and polymeric AMP nanoparticles and coatings.

Liposomes are vesicle bilayers composed of natural phospholipids and cholesterol with an aqueous core (Figure 2). Hydrophilic drugs can be loaded into the aqueous core, while hydrophobic drugs can be incorporated into or adsorbed to the lipid bilayer. Conventional liposomes have shown to successfully reduce toxicity and improve cellular and tissue uptake of AMPs and increase their biodistribution in vivo. However, these carriers are prone to clearance from the bloodstream via opsonization by plasma components and uptake via mononuclear phagocytes [36]. In addition, hydrophobic and electrostatic interactions between cationic AMPs and liposomes can induce membrane deformation and substantial leakage of the liposomes [37,38]. Still, liposomes remain an attractive DDS as their physicochemical and biophysical properties can be easily tuned by modifying the lipid composition or coating of the liposome surface. A variety of AMPs, including colistin, vancomycin, LL-37, indolicin and polymyxin B, have been encapsulated into liposomes (Table 1), indicating the versatility of this DDS. Importantly, liposomes can deliver their AMP into cells mainly through adsorption or endocytosis. A great advantage of liposomes for encapsulation of AMPs in that respect is their enhanced penetration into tissues to target intracellular infections [39].

Liposomes have been evaluated for various applications, including oral, systemic, pulmonary and topical delivery, as well as treatment of intracellular infections. Pulmonary delivery is a favorable application for liposomes, as previous success with pulmonary liposomal formulations has been observed, e.g., for antibiotic-encapsulating systems, such as ARIKAYCE (liposomal amikacin) [40]. As a result, liposomes are also extensively evaluated for the delivery of AMPs. Li et al. showed that colistin-loaded liposomes were equally effective against *Pseudomonas aeruginosa* compared to a colistin solution in vitro. In vivo studies of *P. aeruginosa* tracheal-infection-bearing mice showed that treatment with colistin-loaded liposomes resulted in the survival of 50% of the mice up to 96 h post-infection, while none of the mice treated with empty liposomes or the colistin solution survived longer than 24 h post-infection. In addition, colistin-loaded liposomes reduced systemic exposure of the drug in mice, improving the safety of the AMP [41].

A strategy to enhance the therapeutic efficacy of AMP-loaded liposomes is the modification of the liposomal surface, such as coating with polyethylene glycol (PEG), antibacterial agents or introducing targeting moieties. PEGylation of the liposome surface improves liposome stability, enhances circulation time and reduces uptake by macrophages [42]. Ron-Doitch et al. observed an improvement in bioavailability when using PEGylated liposomes encapsulating LL-37. This DDS showed faster and enhanced uptake of LL-37 by human keratinocyte cells compared to an LL-37 solution. Moreover, the antiviral effect of LL-37 liposomes was enhanced against HSV-1 virus residing in cells, also in a 3D epidermis model [20]. Importantly, the cytotoxicity of LL-37-loaded liposomes was lowered by ~19-fold against keratinocytes upon 24 h incubation compared to LL-37 solution. On the contrary, indolicin-loaded PEGylated liposomes produced by this group were more toxic to keratinocytes upon 24 h incubation than an indolicin solution [20], indicating that the advantages of this DDS may be cargo-specific. Furthermore, liposomes have a negatively charged outer surface, but the surface can be rendered cationic by coating with chitosan or including AMP in the bilayer. Coating with chitosan has been shown to improve the stability of liposomes in the gastrointestinal tract (GIT) and improve absorption of the encapsulated AMP through the epithelium [43]. Chitosan is a cationic polysaccharide biopolymer that is attractive for delivery of antibacterial drugs because the polymer itself has shown antimicrobial activity against clinical isolates of the *Burkholderia cepacia* complex [44] and *Streptococcus mutans* biofilms [45]. Chitosan has also shown biocompatibility and low toxicity [46]. Chitosan has been used by Laverde-Rojas et al. to coat colistin-loaded liposomes. These liposomes were 4-fold more effective against clinical isolates of *P. aeruginosa*, while being equally active against multidrug-resistant strains compared to a colistin solution [47]. Aboumanei et al. executed in vivo biodistribution studies of thigh muscle *Escherichia coli* infection-bearing mice with chitosan-coated liposomes encapsulating colistin and showed that upon oral administration, the bioavailability of colistin in the blood was improved more than 5-fold for chitosan-coated liposomes compared to a colistin solution. In addition, the localization of colistin at the infection site was improved by 125-fold for their formulation 1 h postadministration [48]. Thus, the coating of AMP-loaded liposomes with chitosan increases the effectivity and improves the bioavailability of AMP upon oral administration. Other coatings have also shown success when carefully selected to match the desired application. Menina et al. functionalized colistin-loaded liposomes with extracellular adherence protein (EAP) in order to deliver colistin into epithelial cells. They showed that their coated liposomes enhanced uptake into epithelial cells and more effectively treated *Salmonella enterica* residing in those cells compared to a colistin solution or nonfunctionalized colistin liposomes [22]. A targeted DDS for intravenous administration involving liposomes was described by Jiang et al., who developed a novel red blood cell mimetic hybrid liposome containing polymyxin B and showed that these hybrid liposomes were able to anchor to the membrane of *E. coli*, where it can act as scavenger for bacterial toxins as antivirulence therapy without inducing toxicity. Mice subcutaneously injected with kanamycin-resistant *E. coli* were also most effectively treated by these polymyxin B-loaded hybrid liposomes, increasing survival rates by 50% after 10 days and most effectively reducing bacterial load at the infection site. However, these liposomes were not effective against an *E. coli* intestinal infection in mice when administered orally [49].

Another strategy to enhance therapeutic efficacy of antibiotic-loaded liposomes is coencapsulation of antibiotic with AMP. This can be achieved by decorating the surface of antibiotic-loaded liposomes with AMPs. Wang et al. showed that colistin-coated and ciprofloxacin-loaded liposomes enhanced the killing of *P. aeruginosa* in a time-dependent fashion compared to the use of single compound solutions, indicating that the additive antibacterial activity of colistin and ciprofloxacin was maintained after formulation [50]. Similar cytotoxicity against human epithelial alveolar cells was observed upon 24 h incubation for ciprofloxacin-loaded and colistin-coated liposomes at equivalent concentrations of combinational solution [50]. Likewise, Yu et al. showed that their colistin-coated and ciprofloxacin-loaded liposomes maintained additive antimicrobial activity over time against *P. aeruginosa*, comparable to combinations in solution [51]. Similar to Wang et al., Yu et al. also observed comparable cytotoxicity against epithelial alveolar cells upon 24 h incubation for ciprofloxacin-loaded and colistin-coated liposomes. For human lung cancer cells, no significant cytotoxicity was observed for these liposomes, while the combinatory solution showed significant cytotoxicity at this concentration [52]. Both groups demonstrated a reduced transport capability of the two drugs across human lung cancer cells for these liposomes and showed that the liposomes were trapped in the mucus or adhered to the cell monolayer [52,53]. This allows for a prolonged retention and sustained release of these therapeutic agents at the site of airway infection. Similarly, Faya et al. demonstrated enhanced antimicrobial activity against methicillin-resistant *Staphylococcus aureus* (MRSA) for vancomycin-loaded liposomes decorated with AMP2 or AMP3 compared to the individual compounds. Their formulation also enhanced the eradication of intracellular MRSA in human embryonic kidney cells compared to a vancomycin solution. No hemolysis was observed for both their liposomes and AMP solutions upon 30 min incubation with red blood cells of sheep [23]. The beneficial effect of coformulation was less apparent for the azithromycin-loaded liposomes decorated with DP-7 developed by Liu et al., which were shown to maintain or only slightly increase antimicrobial activity against a panel of Gram-positive and Gram-negative bacterial strains. Azithromycin-loaded and DP-7-coated liposomes showed negligible cytotoxicity towards human cell lines upon 24 h incubation for azithromycin, while the azithromycin solution was slightly more toxic at the same concentration, and upon IV administration in mice, hepatic and renal function and blood system were not affected. Finally, BALB/c mice were infected with MRSA via intraperitoneal injection and 1 h post-infection treated intravenously at the tail with azithromycin and/or DP-7 liposomes and it was shown that all liposomes were able to reduce bacterial burden, but especially the combination of both compounds in the liposomes most effectively reduced bacterial burden [54]. To summarize, the encapsulation of AMPs in liposomes maintained or improved antimicrobial activity, reduced cytotoxicity and enhanced intracellular uptake in vitro, while in vivo liposomes improved targeting, bioavailability and antimicrobial activity.

#### 3.1.2. Niosomes

Niosomes are liposomal vesicles composed of nonionic surfactants, such as polyglycerol alkyl ethers, cholesterol and lipids (Figure 2). Because of the material used, niosomes are more stable than liposomes and the production is more cost-effective [55]. Chauhan et al. produced anionic niosomes encapsulating polymyxin B, which maintained in vitro antibacterial activity against *P. aeruginosa* compared to a polymyxin B solution (Table 1). Ex vivo toxicity against liver cells obtained from sacrificed rats was similar for these niosomes compared to a polymyxin B solution. Most importantly, pharmacokinetic studies in rats revealed that an oral dose of 2 mg/kg of polymyxin B niosomes enhanced AMP content in intestines of rats and stimulated crossing the GIT through transcytosis of M-cells in the intestine, resulting in systemic circulation of the drug compared to intravenous injection of polymyxin B sulfate [56]. Therefore, niosomes could provide the necessary stability in the GIT for oral delivery of AMPs.

#### 3.1.3. Solid Lipid Nanoparticles

Solid lipid nanoparticles (sLNPs) contain a solid lipid matrix stabilized by surfactants (Figure 2). The first sLNPs were developed in the early 1990s, but recently, at the beginning of 2020, sLNPs became widely known, because they were used to encapsulate mRNA for the COVID-19 vaccines from Moderna and Pfizer [57]. The advantages of sLNPs include the ability to use organic-free solvents, the high drug loading of lipophilic drugs, an enhanced stability, also in the GIT tract, and they can be applied on a large scale. The disadvantages of sLNPs include a tendency for gelation, polymorphic transformations, premature drug release during storage, and in some cases, low drug encapsulation due to the crystalline structure of solid lipids [58,59].

A range of AMPs have been encapsulated in sLNPs (Table 1). Ryan et al. produced sLNPs containing lacticin 3147 in order to shield this AMP from degrading enzymes in the GIT and as a potential release system for oral delivery. Preliminary data from zone inhibition tests with *Listeria monocytogenes* showed an improved antimicrobial activity of the sLNPs compared to a drug solution. These sLNPs maintained their bactericidal property in the presence of the enzyme α-chymotrypsin, although to a lesser extent [60]. Severino et al. produced sLNPs containing polymyxin B and showed that these formulations were bactericidal against six AMR strains of *P. aeruginosa* [61]. Fumakia et al. developed sLNPs containing both LL-37 and elastase inhibitor serpin A1 for the treatment of chronic wounds. In vitro studies showed that the dual-loaded sLNPs increased antimicrobial activity against *E. coli* and MRSA compared to single-loaded sLNPs and greatly reduced cytotoxicity against human foreskin fibroblasts and primary human epidermal keratinocytes compared to single drug solutions. In addition, dual-loaded sLNPs reduced wound healing time, induced anti-inflammatory activity and showed sustained permeation across ex vivo rabbit skin [62]. Thus, the encapsulation of AMPs in sLNPs could provide the enhanced stability necessary for oral delivery, but its use could also be extended to other applications, such as topical treatment.

#### 3.1.4. Nanostructured Lipid Carriers

Nanostructured lipid carriers (NLCs) are produced by mixing solid and liquid lipids, leading to special nanostructures in the matrix (Figure 2). NLCs have been developed as second-generation lipid carriers to resolve problems associated with sLNPs, such as limited drug loading and drug expulsion during storage. The advantages of NLCs include a higher encapsulation, lower drug release during storage and improved permeability and bioavailability [58,63].

Various AMPs have been encapsulated into NLCs, including colistin, LL-37 and polymyxin B (Table 1). Sans-Serramitjana et al. compared the antimicrobial activity of SLNPs with NLCs, both encapsulating colistin. Importantly, they showed that colistin-loaded NLCs were more stable compared to sLNPs and retained their bactericidal activity for up to nine months of storage at different temperatures. Moreover, colistin-loaded NLCs maintained antibacterial activity against planktonic bacteria of *P. aeruginosa* and improved activity by approximately 10-fold against biofilms when compared to a colistin solution [64]. A follow-up study showed that colistin-loaded NLCs eradicated biofilm more rapidly and killed bacteria in the inner fraction of the biofilm more effectively compared to a colistin solution. Both effects could be explained by improved penetration of the drug into deeper layers of the biofilm upon formulation [65]. Moreover, Pastor et al. prepared colistin-loaded NLCs for the treatment of multidrug-resistant *P. aeruginosa*. In vitro, activity against planktonic bacteria was reduced up to 16-fold compared to a colistin solution; however, a similar effectivity was reached for colistin-loaded NLCs at 9-fold (pulmonary route) and 24-fold (IM route) lower concentrations compared to a colistin solution in an in vivo acute pneumonia model in mice caused by *P. aeruginosa* [66]. Garcia-Orue et al. investigated the antimicrobial potential of LL-37-loaded NLCs for the treatment of chronic wounds. In vitro studies revealed reduced antimicrobial activity for LL-37-loaded NLCs against planktonic *E. coli* compared to an LL-37 solution and viability of human foreskin fibroblasts was not affected upon 48 h incubation with LL-37-loaded NLCs. Their studies in a full thickness wound model in diabetic mice revealed that topical administration of LL-37 loaded in NLCs resulted in a ~1.5-fold enhanced wound closure compared to an LL-37 solution, increased the reepithelization grade and grade of resolution of inflammation, and thus improved wound healing [67]. Both studies demonstrate that in vitro results are not always a good predictor for in vivo results.

The coencapsulation strategy has also been employed for NLCs, specifically by coating antibiotic-loaded NLCs with AMPs. Rocha et al. prepared dexamethasone-acetate-loaded NLCs coated with polymyxin B and showed that this coformulation increased the MIC against two Gram-negative strains by up to 2-fold and 3-fold, respectively, compared to a polymyxin B solution, without inducing any significant toxic effects against mammalian fibroblasts. Their NLCs were stable at 5 °C for 60 days [68]. Monteiro et al. loaded buparvaquone into NLCs and compared these to NLCs subsequently coated with polymyxin B and/or surface-modified with chitosan or dextran to target macrophages via SIGN-1 or mannan receptor recognition, respectively. The buparvaquone-loaded NLCs without additional coatings maintained bactericidal property against *Leishmania infantum* residing in macrophages and had the highest selectivity index compared to the other formulations, while reducing cytotoxicity against these macrophages by 2-fold compared to the buparvaquone solution [69]. These results highlight the importance of comparative studies in the quest for the most optimal delivery system for AMPs. Overall, it was shown that encapsulation of AMPs into NLCs could improve the eradication of bacteria residing in biofilm and enhance shelf-life stability, while reducing cytotoxicity.

#### 3.1.5. Lipid Nanocapsules

Although promising results have been obtained with liposomes, they have some important drawbacks, including a poor encapsulation of lipophilic drugs, the utilization of organic solvents during production, and an instability in aqueous solutions due to leakage. Therefore, lipid nanocapsules (LNCs) have been developed that have a higher affinity for the encapsulation of lipophilic drugs. These are prepared by a solvent-free method and are stable for >1 year in suspension [70]. Structurally, LNCs are a hybrid of polymeric nanoparticles and liposomes. They consist of an oily core, composed of medium-chain triglycerides, surrounded by a tensioactive and strong membrane, composed of lecithin and PEGylated surfactant (Figure 2). All components are FDA-approved for oral, topical and parenteral administration.

Matougui et al. explored different approaches for the production of LNCs containing AA230, DPK-060 or LL-37 and found that the adsorption strategy effectively increased their encapsulation, maintained or improved their antimicrobial activity against a set of Gram-positive and Gram-negative bacterial strains and enhanced their stability against proteases compared to an AMP solution [71]. Similarly, Groo et al. used reverse micelles to encapsulate AP138 in LNCs and showed that the resulting particles maintained antimicrobial activity, while breakdown by proteases was reduced compared to an AP138 solution [72]. Alternatively, Umerska et al. explored the adsorption of AP114 and AP138 to monolaurin-containing LNCs and found that the adsorption of AMP to monolaurin LNCs resulted in synergy against *S. aureus*, as was confirmed by checkerboard and time-kill assays [73]. Additionally, Rozenbaum et al. observed a synergy for DPK-060 adsorbed to monolaurin LNCs against planktonic and biofilm-residing *S. aureus.* Although to a lesser extent, LL-37 adsorbed to monolaurin LNCs also acted synergistically against *S. aureus* biofilms. Unfortunately, in vivo the synergistic effect was not translated to *S. aureus*-infected wounds on BALB/c mice for DPK-060 LNC, although initial wound healing was enhanced [74]. Still, LNCs could allow for an increased stability of AMP by preventing premature degradation by proteases.

#### 3.1.6. Cubosomes

Cubosomes are highly stable nanoparticles composed of liquid crystalline lipid nanostructures stabilized by a polymer-based outer surface (Figure 2) [75,76]. Different types of inner crystalline structures can be produced, including bicontinuous cubic phase (cubosomes), hexagonal phase (hexosomes) and discontinuous micellar cubic phase (micellarsomes). Compared to liposomes, the inner crystalline structure provides a much higher surface area for AMP loading, while the outer surface still allows for coating or functionalization [76]. The few AMPs that have been loaded in cubosomes are summarized in Table 1. Hankansson et al. encapsulated DPK-060 into LNCs, monolaurin-containing LNCs and cubosomes, loaded these formulations into poloxamer gel as a carrier for topical delivery. DPK-060 formulations in poloxamer gel maintained or decreased antimicrobial activity in vitro and failed to show any beneficial effect compared to DPK-060 poloxamer gel ex vivo and in vivo [77]. In addition, Boge et al. prepared cubic and hexagonal liposomes containing AP114, DPK-060 or LL-37. The in vitro antimicrobial activity of all AMP-loaded hexosomes and LL-37-loaded cubosomes against multiple strains was reduced by more than 2-fold compared to an AMP solution, apart from AP114 and DPK-060-loaded cubosomes, which retained their antimicrobial activity [78]. Boge et al. also showed that the encapsulation of LL-37 in cubosomes protected the peptide from proteolytic enzymes degradation and retained its bactericidal activity against *S. aureus* compared to an LL-37 solution after exposure to enzymes. LL-37-loaded cubosomes initiated no skin irritation in vitro and were effective in an ex vivo wound infection model with pigs; still, the LL-37 solution was more effective [79]. Overall, the encapsulation of AMPs into cubosomes has failed to show an antimicrobial effect exceeding that of a peptide solution so far.

#### 3.1.7. Micelles

Micelles are self-assembled spherical structures composed of single layer lipids that are organized with their hydrophilic head pointing towards the water phase and their hydrophobic tails accumulating in the center of the micelles (Figure 2). Although, micelles are much easier to functionalize compared to liposomes, only hydrophobic AMPs can be loaded into the core of the micelles [80]. Interestingly, the amphiphilicity of some AMPs allows them to spontaneously self-assemble into micelles when in aqueous environment above their critical micelle concentration [81].

The few AMP-loaded micelles described in the literature are summarized in Table 1. Temboot et al. and Madhumanchi et al. prepared polymyxin B-loaded micelles composed of lipid sodium deoxycholate sulfate (SDCS) and showed they maintained antimicrobial activity against planktonic *Acinetobacter baumannii* and *P. aeruginosa* and biofilms of the latter, while reducing cytotoxicity to two types of epithelial cells and reducing the hemolytic activity of polymyxin B [82,83]. Moreover, Kumar et al. encapsulated the aurein-derived AMP peptide 73c in PEGylated phospholipid micelles and showed that peptide-73c-loaded micelles lost in vitro antimicrobial activity against *S. aureus* biofilm, while cytotoxicity was reduced against red blood cells and human peripheral blood mononuclear cells compared to a peptide 73c solution. Surprisingly, the encapsulation of peptide 73c into these micelles resulted in enhanced activity in an in vivo abscess mice model infected with MRSA, compared to other aurein-derived AMP-loaded micelles [84]. Similar to what was reported for the NCLs, these results highlight that in vitro results do not necessarily predict in vivo outcomes. In addition, Zhang et al. prepared self-assembling micelles of DP7 conjugated to cholesterol. Introducing this cholesterol moiety to DP7 resulted in a lower hemolytic activity of these micelles compared to a DP7 solution in vitro and in vivo; in BALB/c mice, DP7 micelles reduced the toxicity against major organs and increased overall survival rates. DP7 micelles were shown to be effective upon IV administration against *P. aeruginosa* infection in zebrafish and MRSA infection in mice. In this murine abdominal infection model, DP7-C also stimulated defensive immune reactions [81]. Overall, it was shown that micelles maintained the antimicrobial activity and simultaneously effectively reduced the toxicity of AMPs.

**Table 1:** AMP encapsulated lipid-based nanoparticles applicable for treatment of various bacterial infections.

Type of Nanoparticles and Particle Composition	AMP	Physicochemical Properties (Size, Surface Charge, Encapsulation Efficiency, Release)	In Vitro and In Vivo Results	Application	Refs.
**Liposomes**					
SCS-Lipoid^®^ S75 SPC liposomes	Colistin	113–137 nm, −66 to −53 mV, EE = 84–92%, 15–43% release in 24 h	- Maintained antimicrobial activity against *P. aeruginosa* */** - Increased lung retention, reduced systemic exposure and enhanced efficacy in pulmonary infection model in mice *	Systemic/pulmonary infections	[41,85]
**Surface-modified liposomes**					
CHL-DSPC-DSPE-mPEG2000 liposomes	LL-37 Indolicidin	107 nm, −2.1 mV, EE = 53% (LL-37) 121 nm, −3.1 mV, EE = 35% (indolicidin)	- Faster and enhanced LL-37 uptake by HaCaT cells * - Reduced cytotoxicity against HaCaT cells/3D Ker-CT model * - Enhanced LL-37 antiviral effect against HSV-1 virus *	Topical/intracellular infections	[20]
CHL-DOPE-lecithin liposomes coated with chitosan	Colistin	485 nm, +5.3 mV	- Antimicrobial activity enhanced against susceptible and maintained against MDR strains of *P. aeruginosa* */** - Empty liposomes have intrinsic antimicrobial activity	Systemic/pulmonary infections	[47]
CHL-S60-lecithin liposomes coated with chitosan	Colistin	156 nm, +16.7 mV, EE = 45–82%, 85% release in 24 h	- Bioavailability improved in thigh muscle infected mice * - Improved localization at the *E. coli*-infected muscle *	Oral delivery/systemic infections	[48]
CHL-DPPC/DSPC-DPPE-GA liposomes coated with EAP	Colistin	203 nm, −15.3 mV, EE = 51%, 20% release in 5 h in PBS or GIT-mimicking media	- Mediated internalization into epithelial cells - Reduced intracellular *S. enterica* load in Hep-2/Caco-2 cells ***	Oral delivery/intracellular infections	[22]
Red blood cell (RBC)-mimetic hybrid liposome composed of lipid S100-DSPE-PEG2000	Polymyxin B	~150 nm, −28 mV	- Prevents hemolysis (neutralization of α-hemolysin and LPS) - Prolonged survival in toxin infected mouse model *** - Only protective at early stage in subcutaneous *E. coli* infection	Antivirulence therapy	[49]
**Coencapsulated liposomes**					
CHL-HSPC-DMPG/DSPG Liposomes	Ciprofloxacin Colistin	~100 nm, anionic, EE = 67% (colistin), EE = 90% (ciprofloxacin), 50–80% release in 30 min then sustained release	- Maintained antimicrobial activity against *P. aeruginosa* * - Similar cytotoxicity against A549 cells * - Reduced transport capacity of drugs across the lung epithelial cell monolayer and enhanced retention on lung surfaces *	Pulmonary infections	[50,53]
CHL-HSPC-DSPG-PEG liposomal powder formulation	Ciprofloxacin Colistin	141–378 nm, −21.0 to −9.2 mV, EE = 47–59% (colistin), EE = 32–71% (ciprofloxacin)	- Maintained antimicrobial activity against *P. aeruginosa* * - Similar cytotoxicity against A459 and Calu-3 cells */** - Reduced transport/enhanced accumulation in Calu-3 cells *	Pulmonary infections	[51,52]
CHL-PC-OA liposomes decorated with AMP2 or AMP3	Vancomycin AMP2/AMP3	137–387 nm, −9.8 to +1.8 mV EE = 27–64%, 49–67% release in 8 h at pH 6, 18–23% release in 8 h at pH 7.4	- Improved antimicrobial activity against MRSA * - No hemolytic activity - Effective against intracellular MRSA	Intracellular infections	[23]
CHL-SPC liposomes incorporating DP7-CHL	Azithromycin DP-7	100–106 nm, +3.7 to +5.3 mV, EE = 97–98% (AZT), DL = 5% (DP7-CHL), ~50% release in 96 h (sustained release)	- Antimicrobial activity against *S. aureus* and *E. coli* maintained * - Slightly reduced cytotoxicity against HEK293 and LO2 cells * - Enhanced antimicrobial effect against MRSA in BALB/c mice ***	Topical infections	[54]
**Niosomes**					
Niosomes composed of Span60 and cholesterol	Polymyxin B	257 nm, −22.5 mV, EE = 72%, stability in SGF (86.22% in SGF pH 1.2 and 78.5% in SIF pH 6.8)	- Maintained antimicrobial activity against *P. aeruginosa* * - Enhanced bioavailability in rat * - No toxicity towards body cells observed in vivo	Oral delivery/intestinal infections	[56]
**Solid lipid nanoparticles (sLNPs)**					
Geleol^TM^-lecithin-Kolliphor^®^ RH40-Transcutol^®^ sLNPs	Lacticin 3147	81–85 nm, EE = 16% (Ltnα), EE = 84% (Ltnβ)	- Improved antimicrobial activity against *L. monocytogenes* * - Improved stability against protease α-chymotrypsin *	Oral delivery/intestinal infections	[60]
Crodacol^®^ CS90/Crodacol^®^ C90-Lipoid^®^ S75 SPC sLNPs complexed with sodium alginate	Polymyxin B	203–574 nm, −40.7 to −24.1 mV, EE = 93–94%	- Maintained antimicrobial activity against resistant strains of *P. aeruginosa* * - Crodacol CS90 lipid also antimicrobial	Topical infections	[61]
Glyceryl monostearate-PC-PVA sLNPs	LL-37 Serpin A1	210–232 nm, −20 to −16 mV, EE = 82–89%, 14% release in 24 h followed by slow sustained release over 15 days	- Improved antimicrobial activity against *S. aureus* and *E. coli* */** - Reduced cytotoxicity against BJ fibroblast cells and keratinocytes */** - Promotes wound healing in vitro	Topical infections	[62]
**Nanostructured lipid carriers (NLCs)**					
Precirol^®^ ATO 5-Miglyol 812-Polysorbate 80-Poloxamer 188 NLCs	Colistin	300–427 nm, negatively charged, EE = 80–95%, sustained release with >50% release in 24 h	- Maintained antimicrobial against planktonic *P. aeruginosa* and killed *P. aeruginosa* in biofilm more rapidly * - More effective killing of bacteria in inner part of biofilm *	Pulmonary infections/biofilm removal	[64,65]
Precirol^®^ ATO 5-Miglyol 182 N/F-Tween^®^ 80-Poloxamer 188 NLCs	Colistin	354 nm, −20.4 mV, EE = 95%, 80% release in 5 h and 92% in 24 h	- Antimicrobial activity reduced against MDR/XDR *P. aeruginosa* * - Same effectivity at lower concentrations in BALB/c mice *	Systemic/pulmonary infections	[66]
Precirol^®^ ATO 5-Miglyol 812N-Tween^®^ 80-Poloxamer 188 NLCs	LL-37	274 nm, −31.6 mV, EE = 96%, DL = 17%	- Reduced antimicrobial activity against *E. coli* * - No cytotoxicity against human foreskin fibroblasts - In vivo, wound healing significantly improved *	Topical infections/chronic wounds	[67]
**Coencapsulated NLCs**					
CP-CCP-Lipoid^®^ S100 SPC-PL-SLS NLCs coated with polymyxin B	Dexamethasone acetate Polymyxin B	231–256 nm, −2.1 to +3.5 mV, EE = 94% (dexamethasone acetate), EE = 99% (polymyxin B coating)	- MIC enhanced against *P. aeruginosa* and *B. bronchiseptica* * - No cytotoxicity against mammalian fibroblast cells	Ocular infections accompanied by inflammation	[68]
Softisan154-MCT-Kolliphor^®^ P188 NLCs coated polymyxin B and surface modified with chitosan or dextran	Buparvaquone Polymyxin B	184 nm, −20.1 mV (BPQ-NLC-PB-[chitosan]); 209 nm, +31.1 mV (BPQ-NLC-PB-[dextran]); 172 nm, −30.9 mV (BPQ-NLC), EE = 99.3–99.7% (BPQ)	- Coating and decorating with PB improved MIC values against *L. infantum* infected microphages (~2-fold) * - Coating and decorating with PB increased cytotoxicity against macrophages (3-fold for chitosan, 70-fold dextran) *	Intracellular infections	[69]
**Lipid nanocapsules (LNCs)**					
Labrafac WL1349-Lipoid S75-Kolliphor HS15-NaCl LNCs (adsorption strategy)	AA230 DPK-060 LL-37	60–77 nm, −3.7 to −0.8 mV, EE = 26–35%	- Antimicrobial activity maintained or improved against strains of *S. aureus*, MRSA, *P. aeruginosa*, *E. coli* and *A. baumannii* * - Stability against proteases improved *	MDR infections	[71]
Reverse micelles in Oleic Plurol-Kolliphor HS-15-Labrafac WL 1349-NaCl-DSS LNCs	AP138	63 nm, −25.6 mV, EE = 98%, 50% release in 2 h, 100% release in 24 h	- Maintained antimicrobial activity against *S. aureus*/MRSA * - Partial protection against protease trypsin	Topical infections	[72]
ML-Solutol^®^ HS15-Labrafac^®^ WL1349-NaCl LNCs	AP114 AP138	36–37 nm, AE = 34–62%, DL =1–3%	- Maintained potent bactericidal activity against *S. aureus* * - Synergism with ML-LNC against MRSA/MSSA	Topical infections	[73]
ML-Solutol^®^ HS15-Labrafac^®^ CC-NaCl LNCs	DPK-060 LL-37	32–135 nm, +5 to +20 mV, AE = 28–42% (DPK-060), AE = 72–77% (LL-37), sustained release	- Synergy for both AMPs against *S. aureus* biofilms - DPK-060 LNCs provided faster initial wound healing in mice *	Topical infections	[74]
**Cubosomes**					
Capmul-90 EP/NF cubosomes in Poloxamer 407 gel	DPK-060	200–300 nm (cubosomes), 50–70% release cubosomes in 24 h	- Maintained bacterial activity against *S. aureus* * - No toxicity observed in skin irritation test	Topical infections	[77]
GMO-Lutrol F127 cubosomes and GMO-OA-Lutrol F127 hexasomes	AP114 DPK-060 LL-37	87–111 nm, −11.1 mV and EE = 27% (AP114), +0.9 mV and EE = 50% (DPK060), +4.5 mV and EE = 81% (LL-37)	- AP114/DPK-060 in cubosomes maintained bactericidal activity against *S. aureus*, MRSA, *P. aeruginosa*, *E. coli* and *A. baumannii* * - Bactericidal activity lost in hexasomes *	MDR infections	[78]
GMO-Poloxamer 407 cubosomes	LL-37	130 nm, no release in 24 h	- Reduced antimicrobial activity against *S. aureus* and *E. coli* * - Protected LL-37 from proteolysis	Topical infections	[79]
**Micelles**					
SDCS micelles	Polymyxin B	126–189 nm, −7.4 to −4.9 mV, EE = 48–57%, >80% release in plasma in 24 h, sustained release	- Maintained antimicrobial activity against planktonic *A. baumannii* and planktonic/biofilm *P. aeruginosa* * - Reduced hemolysis and cytotoxicity towards kidney cells *	MDR Gram-negative infections	[82,83]
DSPE-PEG2000 micelles	Aurein-derived AMPs	12–14 nm	- Reduced hemolysis and cytotoxicity against PBMCs * - Enhanced activity in in vivo MRSA abscess mouse model **	Topical infections	[84]
DP7-CHL micelles	DP7	36 nm, +43.8 mV	- Reduced toxicity and increased survival rates in BALB/c mice * - Effective against *P. aeruginosa* and MRSA infection in vivo - Immunomodulatory effects of DP7-C micelles in mice	Systemic infections	[81]

Acronyms: AE = adsorption efficiency, AMP = antimicrobial peptide, CCP = capric caprylic triglycerides, CHL = cholesterol, CP = cetyl palmitate, DL = drug loading, DMPG = 1,2-dimyristoyl-sn-glycero-3-phosphoglycerol, DOPE = 1,2-dioleoyl-sn-glycero-3-phosphoethanolamine, DPPC = 1,2-di-O-palmitoyl-sn-glycero-3-phosphocholine, DSPC = 1,2-distearoyl-sn-glycero-3-phosphocholine, DSPE-mPEG2000 = N-[carbonyl-methoxypolyethyleneglycol-2000]-1,2-distearoyl-sn-glycero-3-phosphoethanolamine, DSPE-PEG2000 = 1,2-distearoyl-sn-glycero-3-phosphoethanolamine-N-methoxy-polyethylene glycol 2000, DSPG = distearoyl-sn-glycero-3-phosphoglycerol, DSS = dioctyl sodium sulfosuccinate, EE = encapsulation efficiency, HSPC = hydrogenated soybean phosphatidylcholine, MCT = medium chain triglycerides, MDR = multidrug-resistant, ML = monolaurin, GMO = glycerol monooleate, OA = oleic acid, PC = phosphatidylcholine, PEG = polyethylene glycol, PL = polyoxyethylene-polyoxypropylene block copolymer, S60 = Span60, SCS = sodium cholesteryl sulphate, SDCS = sodium deoxycholate sulphate, SGF = simulated gastrointestinal fluid, SLS = sodium lauryl sulfate, SPC = soybean phosphatidylcholine, XDR = extensively drug-resistant; * compared to peptide solution, ** compared to empty nanoparticles or no treatment, *** compared to nonfunctionalized nanoparticles.

### 3.2. Polymeric Nanoparticles

Synthetic biopolymers are commonly utilized for the fabrication of polymeric nanoparticles to improve the efficacy and reduce the toxicity of AMPs. Polyesters, e.g., poly(lactic acid) (PLA) and poly(lactide-*co*-glycolide) (PLGA), are a subtype of synthetic biopolymers characterized by hydrophilic and hydrophobic blocks of chemically different polymer units linked together by covalent bonds that undergo phase separation, which results in the formation of polymeric nanoparticles (NPs) [86]. Polyesters are commonly used to form self-assembled solid or micellar-like polymeric NPs composed of a hydrophobic core and a hydrophilic polymeric shell (Figure 2). Polyesters are ideal building blocks to formulate NPs for biomedical applications due their biocompatibility, biodegradability and favorable safety profile. Additionally, polyesters are commercially available in a range of chemical compositions, molecular weight, and side-chain group. Hence, polyesters offer versatility, flexibility, and the possibility for modified derivatives compared to natural biodegradable biopolymers. Moreover, other subtypes of synthetic biopolymers have been used for the encapsulation of AMPs. For instance, polyanhydrides are a desirable polymer for DDSs as they offer a shorter release profile than polyester-based nanoparticles [87]. Additionally, polysaccharides are frequently used polymers, as they are natural biopolymers composed of monosaccharide units linked by glycosidic bonds [88]. Polysaccharide-based NPs are the second most commonly used type of polymeric nanoparticles for the encapsulation of AMPs, due to their biodegradability, biocompatibility, low-cost and wide availability from a range of resources. In nature, polysaccharides can be extracted from animal (e.g., chitosan), plant (e.g., pectin), and algal origin (e.g., alginate) [89]. Moreover, polysaccharide NPs can be divided into positively charged oligosaccharides (e.g., chitosan) and negatively charged polysaccharides (e.g., pectin, alginate). An overview of AMP-encapsulating solid nanoparticles formed using synthetic and natural polymers is provided below.

#### 3.2.1. Synthetic Nanoparticles

##### PLGA Nanoparticles

PLGA is of particular interest for AMP delivery and tissue regeneration purposes as lactate, a breakdown product of PLGA, is known to accelerate neovascularization and enhance wound healing [90]. Additionally, simple variations of the polymer type (e.g., molecular weight, end-capping group or polymer ratio) can fine tune the encapsulation of AMP and its release profile. Due to these attributes in combination with it being commercially available and FDA-approved, PLGA has been extensively used for the controlled release of AMPs (Table 2). Casciaro et al. found that PLGA encapsulation of frog skin AMP esculentin was able to enhance the antimicrobial activity of the peptide in a mouse model of acute *P. aeruginosa* lung infection by up to ~17-fold compared to the peptide solution [91]. Chereddy et al. showed accelerated wound closure in an in vivo splinted mouse full thickness model with PLGA-LL-37 in a dose-dependent manner, whereas the peptide solution showed no change in activity at higher doses. However, PLGA-LL-37 showed ~25% in vitro killing activity compared to ~50% with the LL-37 solution against *E. coli* [90]. Additionally, Vijayan et al. demonstrated the therapeutic potential of a dual drug delivery system composed of the AMP K4 conjugated to PLGA-encapsulated growth factors [92]. The peptide-conjugated NPs demonstrated broad-spectrum antimicrobial activity against both Gram-positive and Gram-negative bacteria and promoted the migration of cell monolayers in an in vitro wound healing assay 24 h post-treatment. Furthermore, Gomez et al. showed the encapsulation of synthetic AMPs, namely GIBIM-P5S9K (G17) and GAM019 (G19), in PLGA resulted in a 3-fold and 1-fold decrease of the minimum inhibitory concentration to inhibit 50% of bacterial growth (MIC_50_) of MRSA for G17NPs and G19NPs, respectively; and the MIC_50_ of *E. coli* showed a 2-fold decrease for both formulations 8 h post-treatment [93]. Additionally, Sharma et al. demonstrated enhanced in vitro killing of *E. coli* with PLGA-encapsulated HHC10 in a concentration-dependent manner and higher cellular uptake in mouse macrophages compared with a peptide solution 12 h post-treatment [94]. The bactericidal activity of PLGA-encapsulated HHC10 occurred via multimodal interactions with bacteria, which involved cell-membrane lysis on direct interaction with bacteria, and activation of apoptotic death pathway (cathepsin-B) upon internalization in *E. coli*-infected cells in vitro. HHC10 showed less cytotoxic effects on mouse macrophages compared to a peptide solution at equivalent amounts 24 h post-treatment. These findings suggest that incorporation of HHC10 in PLGA could reduce the probability of inducing the membrane lysis of mammalian cells due to the higher intracellular uptake observed compared with a peptide solution. Sharma et al. functionalized PLGA NPs, encapsulating IDR-1018 with a biofilm penetrating ligand, N-acetyl cysteine (NAC), to improve their antibiofilm activity against *Mycobacterium tuberculosis* [21]. The results showed that NAC-coated NPs exhibited a significantly increased disruption of biofilm in vitro and a ~1.6-fold increase in cellular uptake by *M. tuberculosis*-infected macrophages compared to nonfunctionalized PLGA NPs. NAC-coated PLGA NPs displayed a significantly higher bacterial killing activity than noncoated PLGA NPs, which is attributed to higher uptake of NAC-PLGA NPs combined with additive antibacterial activity of the NAC ligand.

##### PLA Nanoparticles

PLA is another important synthetic polymer commonly used for biomedical application due to its biocompatibility, tailorable properties and established fabrication techniques. The chemical structure of PLA consists of monomer units connected by ester bonds, which are naturally degraded by hydrolysis [95]. Cruz et al. performed a comparative study evaluating the antimicrobial activity of PLA- and PLGA-encapsulated peptides versus a peptide solution (GIBIM-P5S9K) using the in vitro broth microdilution method [96]. They showed a significant reduction in the MIC_50_ with peptide-loaded PLA/PLGA NPs (MIC_50_ = 0.5 µM) compared with the peptide solution (MIC_50_ = 10 µM) against *E. coli*, *S. aureus* and *P. aeruginosa* 8 h post-treatment. Moreover, there were no significant differences in antibacterial activity between the two types of polymeric nanoparticles, apart from killing against *E. coli* where PLA antimicrobial activity was higher (MIC_50_ < 0.5 µM) compared to PLGA nanoparticles (MIC_50_ = 1–10 µM). The higher antimicrobial activity observed with encapsulated peptide could be mediated by electrostatic interactions between the positively charged PLA/PLGA nanoparticles (20–30 mV) and negatively charged surface of Gram-negative and Gram-positive bacteria, which may lead to a higher local peptide concentration on the surface of bacteria [97,98].

##### Poly(l-lactic acid-*co*-d,l-mandelic acid) Nanoparticles

Although AMP-loaded PLGA and PLA DDSs show both structural stability and antimicrobial activity, the initial burst release is undesirable for clinical application due to possible side effects, which may reduce their clinical usability. Additionally, the sustained release phase that follows the burst phase might not achieve the MIC needed to kill bacteria. Polyanhydrides, such as poly(l-lactic acid-*co*-d,l-mandelic) acid nanoparticles (poly(LA-*co*-MA) NPs) are a class of biodegradable polymers in which the polymer backbone is composed of repeating units connected by anhydride bonds. The degradation of polyanhydrides consists of two stages with an initial swelling at the surface of the matrix without degradation (induction period), followed by an erosion of oligomers by swelling of the main chain [99]. The degradation products are nontoxic and the release profile of polyanhydrides is almost zero-order and easily adjustable, usually 1 to 14 days, making them ideal candidates for the fabrication of controlled release systems [87]. Wang et al. encapsulated cathelicidin-BF-30 in poly(LA-*co*-MA) NPs with a diameter of around 275 nm and an encapsulation efficiency and loading capacity of 92% and 8%, respectively. They showed poly(LA-*co*-MA)-encapsulated cathelicidin-BF-30 had an antibacterial activity profile similar to a peptide solution on several bacterial species, including *E. coli* and *S. aureus*, using the in vitro zone inhibition test [100]. Additionally, they showed that the encapsulated peptide had less than 5% hemolysis and no cytotoxicity to HEK293 cells up to 150 µg/mL, indicating that poly(LA-*co*-MA) NPs could be a potential drug carrier for AMPs.

#### 3.2.2. Natural Nanoparticles

##### Chitosan Nanoparticles

Chitosan is a natural biodegradable and biocompatible polymer that can provide controlled release, improved bioavailability and safety of AMPs. Chitosan nanoparticles have been proven to effectively enhance cellular penetration, intracellular retention and subcellular distribution of AMPs [101]. Moreover, chitosan has intrinsic bactericidal activity that can be exploited to improve the antibacterial properties of AMPs. For these reasons, chitosan is commonly used for the delivery of several AMPs (Table 2). Sun et al. compared the antibacterial effects of king-cobra-derived OH-CATH30 (OH30) peptide loaded in chitosan and poly-γ-glutamic acid composite (CMCS-OH30) NPs in a full-thickness excision model in vivo [102]. Mice treated with CMCS-OH30 NPs healed faster with 69% wound closure compared to a combined range of 36–58% attained with no treatment, a peptide solution and empty CMCS NPs 5 days post-treatment. In terms of antimicrobial killing activity, CMCS-OH30 NPs were very effective with 100% in vitro killing against *E. coli* at all time intervals for up to 24 h. Moreover, Li et al. showed prolonged antimicrobial and inhibitory effects of KSL (KKVVFWVKFK-CONH_2_) peptide formulated into PLGA/chitosan composite microspheres on oral bacteria (*F. nucleatum*) for up to 80 days post-treatment using the inhibition zone assay [103]. Chitosan was chosen due to its positive charge in order to neutralize the acidic degradation products of PLGA (glycolic acid and lactic acid). These data demonstrate that the constructed chitosan-based NPs play a significant role in promoting antibacterial and wound healing effects.

##### Pectin Nanoparticles

Pectin is a naturally occurring polysaccharide consisting of mainly D-galacturonic acid units joined by glycosidic linkage units [104]. Pectin is extracted from apple and citrus peel and is commonly used in the food industry as a thickening agent. However, pectin can also be exploited for controlled drug delivery due to its excellent biocompatibility, low production costs and unique properties. For instance, pectin can easily adhere to mucosal surfaces which can promote the retention time of AMPs and it can also become easily degraded by microbial enzymes [105]. Krivorotova et al. demonstrated the antimicrobial activity of nisin-loaded nanoparticles in vitro against two Gram-positive (*Arthrobacter* sp. and *Bacillus subtilis*) and two Gram-negative bacteria (*E. coli* and *Klebsiella* spp.) using the agar-diffusion assay [106]. They showed that the nisin-loaded pectin NPs had a higher antimicrobial activity against Gram-positive compared to Gram-negative bacteria. Moreover, nisin-loaded pectin NPs were more than 100-fold more effective compared to sodium benzoate, a conventional preservative, in the killing of Gram-positive and Gram-negative bacteria. These findings suggest that nisin-loaded pectin nanoparticles are a suitable polymeric antimicrobial delivery system for the delivery of AMPs.

#### 3.2.3. Future Perspective of Polymeric Nanoparticles

A major drawback of nontargeted polymeric nanoparticles is the lack of chemical functionalities on the surface to induce site-specific interactions, such as adhesion, internalization and penetration through biological membranes. Additionally, delivery of hydrophilic molecules (e.g., peptides, proteins) is limited by poor encapsulation and a large burst release of encapsulated drug within the first few hours. The initial burst release is mainly due to desorption of hydrophilic AMPs from the surface of the biomaterial devices composed of hydrophobic polymers [107], due to poor associations between the drug and the polymer [108]. Moreover, hydrolysis of polymers, namely PLGA and PLA, can result in peptide/protein aggregation and denaturation due to the local acidic microenvironment, and hence restrict its applications [109].

The functionalization of polyester-based therapeutics is of particular interest because it allows the fine tuning of particle characteristics, such as hydrophilicity, surface charge and release at the site of infection. This can enhance targeted drug delivery of AMPs, for example by promoting intracellular drug delivery of AMPs and also allowing accumulation at infection sites. Furthermore, an additive or even synergistic antimicrobial effect can be achieved by encapsulating the AMP in a polymer with intrinsic antimicrobial properties, such as chitosan. Functional groups can be incorporated on the aliphatic backbone of the polyesters via direct conjugation or with additives during the formulation process to elicit specific drug or cell interactions. Polymeric drug delivery systems have unique properties and functions which make them suitable for AMP delivery, though certain issues related to stability, toxicity and loading/encapsulation need to be addressed by modifying the formulation process for effective AMP delivery. To date, there are no marketed polymeric nanoparticle formulations for AMPs.

**Table 2:** AMP-encapsulated polymeric nanoparticles applicable for treatment of various bacterial infections.

Type of Nanoparticles and Particle Composition	AMP	Physicochemical Properties (Size, Surface Charge, Encapsulation Efficiency, Release)	In Vitro and In Vivo Results	Application	Refs.
**Synthetic AMP-loaded nanoparticles**					
PLGA	Esculentin	261–282 nm, −0.7 to −0.8 mV, EE = 100%, LC: 2%, 60% released after 3 h, then sustained for 3 days	- Enhanced antimicrobial activity in vitro and in vivo *against* a mouse model of acute *P. aeruginosa* after 36 h */** - Nanoencapsulation lead to a 3-log reduction of pulmonary *P. aeruginosa* growth for up to 36 h in vivo */**	Systemic/lung infection	[91]
PLGA	LL-37	304 nm, −21 mV, EE = 70%, LC = 1%, ~40% burst release, then 14 day sustained release	- Accelerated wound healing in excisional wounds in vivo */** - Enhanced antimicrobial activity against *E. coli* in vitro */**	Topical/wound infection	[90]
PLGA	G17 and G19	284–291 nm, +7.3 to +12.9 mV, EE = 90%, LC: 0.6–0.9%, 45% released after 1 h, controlled release up to 48 h	- Decreased MIC50 against MRSA in vitro */** - Encapsulation of peptides in PLGA decreased the MIC50 for up to 4 times against *E. coli* in vitro */**	Topical/wound infection	[93]
PLGA	HHC10	320 nm, +13.3 mV, EE = 54%, 42% release up to 10 h followed by plateau phase	- Maintained inhibition of *E. coli* growth in vitro ** - Nontoxic to macrophage mouse cells in vitro after encapsulation - 91% maximum cellular internalization in 24 h **	Systemic infection	[94]
PLGA with N-acetylcysteine coating	IDR-1018	5.1–6.2 μm, EE = 59–62%, sustained release for up to 48 h followed by a controlled release of peptide for up to 120 h	- Coated and IDR1018-loaded PLGA nanoparticles reduced *M. tuberculosis* load in macrophage cultures in vitro */** - Significantly reduced lung inflammation in vivo in *M. tuberculosis* infected mice */**	Systemic/lung infection	[21]
PLGA	K4	416 nm, +1 mV, 89% peptide conjugation	- Peptide conjugation to PLGA NPs reduced killing activity against *S. aureus* and *P. aeruginosa* */**	Topical/chronic wound infection	[92]
PLGA	Plectasin	215 nm, −18 mV, EE: 71–90%, 77% release after 1 h, rest was released over 24 h	- Encapsulated peptide enhanced the in vitro antimicrobial activity against *S. aureus* */**	Systemic infection	[110]
PLGA/PLA	GIBIM-P5S9K	258–352 nm, +22.7 to +29.4 mV, EE = 55–75%, 50% peptide release after 8 h and a successive slower release phase	- Peptide loaded nanoparticles demonstrated enhanced antibacterial activity against *E. coli*, *MRSA*, and *P. aeruginosa* in vitro */**	Topical infection	[96]
Poly(LA-*co*-MA)	BF-30	2.75 µm, EE = 92%, LC = 8%, no initial burst release, only controlled release of peptide was observed after 25 days	- Released peptide inhibited growth of *E. coli*, *S. aureus*, *S. typhi*, *B. subtilis* in vitro against *F. nucleatum* using the inhibition zone assay * - Did not significantly enhance the antimicrobial effect *	Topical infection	[100]
**Natural AMP-loaded nanoparticles**					
PLGA-chitosan composite	KSL	61–67 µm, EE = 70–93%, LC = 1.7–3.7%, 25–35% released after 10 days, and 80–90% released after 80 days	- Encapsulation of KSL peptide enhanced antimicrobial activity in vitro against *Fusobacterium nucleatum* using the inhibition zone assay **	Topical infection in oral cavity	[103]
Carboxymethyl chitosan	OH-CATH30	258 nm, 30.2 mV, EE = 82%, LC = 33%, near-linear release with 70% released at 24 h	- 100% killing of *E. coli* in vitro over 24 h compared to ~25% ** - Significantly enhanced wound healing in vivo in a mouse model */**	Topical/skin infection	[102]
Pectin	Nisin	200–500 nm, −20–−45 mV, EE = 100%	- Nisin-loaded pectin NPs were 100-fold more effective compared to food preservative, sodium benzoate in vitro	Food preservation	[106]

Acronyms: AMP = antimicrobial peptide, EE = encapsulation efficiency, LC = loading capacity, MIC_50_ = minimum inhibitory concentration to inhibit the growth of 50% of organisms, PLA = poly (lactic-*co*-acid), PLGA = poly (lactic-*co*-glycolic acid), Poly(LA-*co*-MA): poly(L-lactic acid-*co*-D,L-mandelic acid); * compared to peptide solution ** compared to empty nanoparticles or no treatment.

### 3.3. Polymeric Nanogels

In recent years, nanogels have received increased attention as a versatile delivery system due to their unique potential resulting from the combined features of hydrogels and nanoparticles. These soft nanoparticles are three-dimensional cross-linked polymeric networks made of water-soluble natural or synthetic polymers that have the ability to absorb high amounts of water or biological fluids into the formed network while maintaining their structure (Figure 2) [111,112]. The high hydrophilicity is due to the abundance of hydrophilic groups, such as –OH, –CONH–, –CONH_2_– and –SO_3_H on the polymer backbone [113], which provides a high biocompatibility and allows a high encapsulation of peptides and proteins [114]; nanogels avoid clearance by phagocytic cells, allowing both passive and active drug targeting [115]. The preparation of particles in mild conditions without the need for organic solvents makes nanogels very desirable drug carriers for biomacromolecules. Nanogels can protect the encapsulated peptide from proteolytic degradation and reduce toxicity without affecting the antimicrobial activity. Drug release from nanogels can occur in response to a wide variety of environmental stimuli, such as ionic strength, pH and temperature [113,116]. The drug diffusion out of a hydrogel matrix is primarily dependent on the mesh sizes within the matrix of the gel [117], which, in turn, is affected by several parameters, including, mainly, the degree of crosslinking, chemical structure of the composing monomers and when applicable, the type as well as intensity of the external stimuli. These parameters can be tailored to achieve the desired rates of macromolecular diffusion [111,112]. Typically, nanogels show a very fast release of biomacromolecules, within 10–72 h.

The low interfacial tension and the deformability of nanogels can potentially minimize nonspecific protein adsorption [111], and improve their penetrating properties across mucus and bacterial biofilms making nanogels ideal nanocarriers for the treatment of biofilm infections. Nanogels have also been formulated into an inhalation powder for lung delivery [118]. Upon pulmonary administration to rats, the nanogels slowly released the encapsulated antimicrobials, resulting in a longer drug residence time in the lungs and decreased levels in other organs, which is expected to reduce side effects associated with the treatment. These findings confirm the significance of the targeting potential and suitability of nanogels for a formulation into final products for administration to patients.

A variety of both natural and synthetic polymers have been evaluated for the controlled release of peptides from nanogel matrices, such as chitosan, hyaluronic acid and alginate or synthetic polymers such as poly (ethyl acrylate-*co*-methacrylic acid) and poly(N-isopropylacrylamide) (PNIPAAm) (Table 3). Both cationic and anionic polymers have been utilized for the preparation of nanogels.

#### 3.3.1. Natural Cationic Polymer-Based Nanogels

Chitosan-based nanoparticles do not self-assemble, instead they form noncovalent interactions with anionic molecules to form polyelectrolyte complexes [119]. This gelation process is due to the formation of inter and intra cross-linkages between polymer chains, mediated by the polyanions [120]. The cationic charge of chitosan allows this polymer to interact with anionic polymers, macromolecules and even with certain polyanions, upon contact in an aqueous environment [120,121]. Chitosan nanoparticles can also be formed upon ionotropic gelation with tripolyphosphate [122]. Piras et al. have shown that chitosan/tripolyphosphate complexes significantly reduced the cytotoxicity of the encapsulated peptide temporin B in mouse embryo fibroblasts, while increasing antibacterial activity, resulting in a 6-fold reduction of bacterial counts after 2 days of exposure to the particles [123]. Rishi et al. utilized a chitosan/tripolyphosphate complex as a delivery system for the peptide cryptdin-2 for oral administration and treatment of murine *Salmonella* infections in vivo [124]. A histological evaluation of small intestine sections showed that treatment with cryptdin-loaded nanoparticles allowed a significant protection from infection, presenting as normal ileum compared to infected but nontreated mice. Encapsulated peptide also prevented the liver toxicity associated with peptide solution treatment, further indicating the improved safety of the formulation. Most importantly, the treatment of infected mice with complex-encapsulated peptide showed 83% survivability compared to 100% mortality in animals treated with a peptide solution.

Utilizing chitosan as the nanogel matrix often provides a positive surface charge, which may increase macrophage uptake, making it a suitable delivery system for treatment of intracellular infections, such as *M. tuberculosis*. Sharma et al. showed that encapsulation of the peptide pep-H in chitosan nanogels resulted in the formation of nanoparticles with a cationic surface charge, which then allowed an 80% reduction of intracellular bacterial load in comparison to equivalent concentrations of peptide in solution, which only showed a 12% reduction in intracellular *M. tuberculosis* load [125]. Chitosan-based nanogels have also demonstrated an increased residence and a close contact with mucosa due to their mucoadhesive property, making them a desirable system for topical mucosal administration [126]. However, the positive charge of chitosan may inhibit efficient binding and encapsulation of cationic AMPs, as is the case for the peptide Pep19–2.5 [127]. For such AMPs, anionic polymers are more suitable.

#### 3.3.2. Natural Anionic Polymer-Based Nanogels

Two most commonly used natural anionic polymers for the formulation of AMPs are hyaluronic acid and alginate [128]. Both polymers have several unique properties that make them desirable to form matrix networks for the encapsulation of peptides and proteins. These include: (a) a relatively inert aqueous environment inside the nanogels; (b) mild, room temperature encapsulation process, free of organic solvents; (c) high gel porosity allowing a diffusion of biomacromolecules out of the matrix; and (d) biodegradability of the polymer under normal physiological conditions. Hyaluronic acid (HA) is a natural, hydrophilic and anionic polymer that can be modified with lipid side chains, which allow physical cross-linking of the polymer chains by self-assembly in water, leading to the formation of nanogels with good loading capability for various therapeutic agents. As HA does not cross-link on its own, a modification of the polymer backbone is required to allow cross-linking of the polymer chains. Water et al. and Klodzinska et al. showed that lipid-modified HA-based nanogels are particularly effective in shielding the cationic charge of the encapsulated peptide and reducing associated cytotoxicity, without reducing the antimicrobial effect of the peptide [129,130]. Klodzinska et al. also observed that encapsulation of a different peptide, DJK-5, into the octenyl-modified HA-based nanogel reduced peptide toxicity in vivo while maintaining the antimicrobial activity [19]. Despite an overall negative or neutral charge, hyaluronic acid-based nanogels have also shown promising results in terms of eukaryote internalization and colocalization with intracellular bacteria. Simonson et al. encapsulated the model peptide green fluorescent protein in poly-l-lysine cross-linked HA nanogels to illustrate rapid internalization of this system in alveolar basal epithelial cells followed by endosomal escape to allow delivery of the drug intracellularly [131]. Silva et al. used lipid-modified HA-based nanogels to deliver the peptide LLKKK18 to *M. tuberculosis* infections in macrophages [132]. The nanogels allowed the delivery of a significantly higher amount of LLKKK18 into macrophages and protected the peptide from enzymatic degradation once internalized. A similar carrier system developed by Montanari et al. proved to be effective in delivering levofloxacin into HeLa cells to treat both *P. aeruginosa* and *S. aureus* intracellular infections [133]. Additionally, Klodzinska et al. have shown that such lipid-modified HA-based nanogels also have very good mucus and biofilm penetration when encapsulating the antibiotic azithromycin [134], well known for binding to mucins, which hinders their activity, indicating their applicability as a delivery system for AMPs [135]. HA-based nanohydrogels have also been used to deliver the enzyme alginate lyase, an enzyme that can degrade alginate, one of the main components of the bacterial biofilm matrix of *P. aeruginosa* [136].

Another natural anionic polymer suitable for the delivery of AMPs is alginate, a naturally occurring polymer derived from brown algae, which is increasingly used in both food and pharmaceutical applications. Alginate can be ionically cross-linked by addition of divalent cations, such as Ca^2+^, Sr^2+^ or Ba^2+^ in aqueous solutions to form soft nanoparticles, whereas monovalent cations, as well as Mg^2+^, do not induce cross-linking [137]. A range of biomacromolecules have been encapsulated in alginate nanogels, including AMPs. Kuhlmann et al. encapsulated the peptide pep19–2.5 in alginate nanogels and showed that this delivery system protected 91% of the encapsulated peptide from degradation in simulated gastric fluid [127]. The antimicrobial activity was shown to be dependent on the release profile, with release occurring rapidly (within 1 h) in biorelevant media. A similar rapid release profile was observed by Borro et al., where polymyxin B was efficiently encapsulated (encapsulation efficiency of >80%) in alginate nanogels at low ionic strength and released completely at physiological osmolarity within 3 h [138]. This is in contrast to the slower release, occurring over 48–72 h, that was observed also for nanogels composed of HA.

#### 3.3.3. Synthetic Polymer-Based Nanogels

A vast range of synthetic polymers for the preparation of drug delivery systems has been developed, though few reports are available regarding the encapsulation of AMPs in such systems. Among synthetic polymers used for this purpose, poly(allylamine)-poly(N-isopropylacrylamide) (PAA-g-PNIPAAm) and poly (ethyl acrylate-*co*-methacrylic acid) have been described. Masuda et al. encapsulated the peptide E5 by forming a polyelectrolyte complex with (PAA-g-PNIPAAm) and found that using the thermoresponsive PNIPAAm side chain allowed a better control of the membrane-disrupting activity of the delivery system on a bacterial membrane model [139]. Nordström et al. found that nanogels composed of poly(ethyl acrylate-*co*-methacrylic acid) protected the encapsulated peptides LL-37 and DPK-060 from degradation by infection-related proteases and in the case of peptide DPK-060, improved the antimicrobial activity of the peptide as determined by MIC [140].

Overall, nanogels composed of both natural and synthetic polymers can be promising carriers if the most suitable system for the required purpose is chosen. Nanogels have demonstrated higher encapsulation efficiency and a desirable fast release profile, which can help achieve fast eradication of the infection without allowing development of bacterial resistance. Nanogels composed of natural biodegradable polymers have proved to be a more promising, viable and safe option for controlled delivery of AMPs in comparison to synthetic polymer-based nanogels, due to their excellent biocompatibility and biodegradability.

**Table 3:** AMP-encapsulated nanogels applicable for treatment of various bacterial infections.

Type of Nanoparticles and Particle Composition	AMP	Physicochemical Properties (Size, Surface Charge, Encapsulation Efficiency, Release)	In Vitro/In Vivo Results	Application	Refs.
**Natural cationic polymer-based**					
Chitosan: tripolyphosphate	Temporin B	185 nm, +8.8 mV, up to 75% EE, burst effect + gradual release (17% over 15 days)	- Increased antibacterial activity and sustained antibacterial action against various strains of *S. epidermidis* for 4 days * - Reduced cytotoxicity towards mouse embryo fibroblasts *	Topical infections	[123]
Chitosan	Pep-H	244 nm, +12 mV, 72% EE, 30% burst release, up to 50% released over 72 h	- H-CSNPs increased efficacy of Pep-H against intracellular *M. tuberculosis* at 5–10 times lower concentration *	Intracellular infections	[125]
Chitosan: tripolyphosphate	Cryptdin-2	105 nm, −22 mV, 60% EE and 65% in vitro release in 4.5 h	- Mice infected with *S. enterica* showed 83% survivability compared to 100% mortality in peptide treated animals	Intestinal infections	[124]
Chitosan and poly-γ-glutamic acid	LL-37	793–2128 nm, −36 to +50 mV, 23–76% EE, 90% released in 10 h	N/A	Infections	[121]
2,3-Dimethyl maleic anhydride grafted chito-oligosaccharide	Polymyxin B	154 nm, −8.7 mV	- Maintained antimicrobial efficacy towards *P. aeruginosa* and *E. coli* and reduced cytotoxicity * - Increased safety in vivo *	Systemic infections	[141]
**Natural anionic polymer-based**					
Alginate	Polymyxin B	100–125 nm, −7 to −35 mV, ~90% EE	- Comparable induction of carboxyfluorescein leakage from bacterial mimicking DOPE/DOPG liposomes *	Infections	[142]
Alginate	Pep19–2.5	342–841 nm, released in pancreatic fluid in 1 h	- Maintained inhibition of cytokine induction *	Gastrointestinal infections	[127]
Octenyl succinic anhydride-modified HA	Novicidin	80–144 nm, −24 to −57 mV, 15–71% EE Complete release over 12 days	- Antimicrobial activity maintained towards *S. aureus* and *E. coli* * - Significantly reduced cytotoxicity for HUVEC and NIH3T3 cells *	Systemic infections	[130]
Octenyl succinic anhydride-modified HA	DJK-5	174–194 nm, −11.6 to −9.5 mV, 33–60% EE, complete release in 48 h	- Maintained bactericidal in vivo in *P. aeruginosa* skin infection * - Significantly reduced toxicity after systemic/subcutaneous administration *	Systemic or topical infections	[19]
Octenyl succinic anhydride-modified HA	LBP-3	155–250 nm, −10 to −28 mV, 37–90% EE	- Improved *P. aeruginosa* killing kinetics * - Decreased cytotoxicity to liver cells *	Systemic or pulmonary infections	[129]
Poly-L-lysine cross-linked HA	Vancomycin GFP	120 nm, −15.4 to −35 mV, DL of 4%, complete release in 48 h	- Internalized by lung cells - Improved antimicrobial activity towards *P. aeruginosa*, *E. coli*, *A. baumannii*, *S. enterica* and *S. aureus* *	Intracellular of pulmonary infections	[131]
Oleyamine-modified HA	Vancomycin	201–360 nm, −17.6 to −20.4 mV, 26–43% EE, drug release over 72 h	- 4-fold lower MIC towards *S. aureus* and MRSA * - Increased impact on MRSA membrane *	Infections	[143]
11-Amino-1-undecanethiol hydrochloride-modified HA	LLKKK18	533 nm, +2.4 mV, approx. 70% EE	- Internalized by macrophages - Colocalized with *M. tuberculosis* and *M. avium* within host cells	Intracellular infections	[132]
PEG-poly(glutamic acid)	MSI-78	80–120 nm, −16 to −38 mV, 75–87% EE, approx. 80% released in 4 days	- Reduced hemolysis * - Antimicrobial activity *E. coli*, *B. subtilis* and *S. aureus* not affected *	Systemic infections	[144]
**Synthetic polymer-based**					
Poly (styrene sulfonate)	Polymyxin B	166–186 nm, −40 mV, approx. 80% released	- Increasing antimicrobial activity towards *P. aeruginosa* with increasing polymyxin B concentration	Infections	[145]
PAA-g-PNIPAAm polyelectrolyte complex	E5	N/A	- E5 peptide in PAA-g-PNIPAAm induced liposome leakage * - More significant leakage at 35 °C than 25 °C	Infections	[139]
PEG20K-hbG3-OH dendritic nanogels	DPK-060 LL-37	205–331 nm, −5 to +5 mV, 40–60 μM/0.1 wt %	- Maintained membrane destabilizing activity * - Reduced cytotoxicity towards erythrocytes * - Maintained antimicrobial activity towards *E. coli* *	Systemic or topical infections	[146]
Poly(ethyl acrylate-*co*-methacrylic acid) microgels	DPK-060 LL-37	50–260 nm, −10 to −30 mV, 35–75% peptide released in 2 h	- Low cytotoxicity towards erythrocytes * - Bacterial-mimicking membrane disruption mediated almost exclusively by peptide release	Systemic infections	[140]
Poly (EA/MAA/BDDA) microgels	EFK17	N/A size, −30 mV, 60–100% release in 1 h	- Antimicrobial activity towards *E. coli* maintained *	Infections	[147]

Acronyms: AMP = antimicrobial peptide, EA/MAA/BDDA = ethyl acrylate/methacrylic acid/1,4-butandiol diacrylate, GFP = Green fluorescent protein, HA = hyaluronic acid, MIC = minimum inhibitory concentration, PAA-g-PNIPAAm = PAA main chain and thermoresponsive poly(N-isopropylacrylamide) graft chains, PEG = polyethylene glycol; * compared to peptide solution.

### 3.4. Polymeric AMP Coatings

Medical devices, such as orthopedic implants and catheters, can improve the quality of lives. Unfortunately, their application can be compromised by their propensity to become colonized by bacteria forming a biofilm. Bacteria released by these biofilms can lead to serious infections, including bloodstream and recurrent urinary tract infections [148,149]. To prevent bacterial colonization, AMPs have been incorporated into or immobilized onto the surfaces of medical devices with mixed success. In recent years, the covalent immobilization of AMPs onto surfaces has steadily developed into the most important strategy to equip medical devices with antibacterial properties [150,151]. This strategy provides a higher AMP surface availability and a more homogeneous distribution over the surface than peptide incorporation or adsorption methods [152]. Moreover, covalent immobilization can protect the peptide from enzymatic degradation, protein binding, and may avoid the toxicity associated with the application of high AMP concentrations. However, the main disadvantage of this strategy relates to its limited antimicrobial effect on bacteria and biofilms in tissues surrounding the medical device. This limitation can be circumvented by a coating that releases the AMP in a controlled fashion. Here, we focus on the antibacterial effects of polymeric AMP coatings on bacteria and bacterial biofilms and indicate if the coating is releasing the AMP in a controlled fashion (Table 4). For the preparation of these coatings, the AMP is often first conjugated onto the polymer either by amide bond formation, click chemistry or immobilized onto the polymer by other chemical reactions, such as Schiff’s base reaction, photo-cross-linking and oxidation. Thereafter, the functionalized polymer may be attached to the surface by chemical reactions, including surface-initiated atom transfer radical polymerization, silanization and electrospinning. Polymeric AMP coatings may be categorized into three main classes based on their structure: (1) thin layer polymer coatings, including self-adhesive polydopamine-based layers, (2) polymeric brushes, and (3) layer-by-layer coatings (see Figure 2).

#### 3.4.1. Thin Layer Polymeric Coatings

The antibacterial effects of the AMPs immobilized in thin layer polymeric coatings are summarized in Table 4. Costa et al. used spin coating to produce a thin film of chitosan on titanium (or gold) as a substrate for AMP immobilization and determined the efficacy of the immobilized AMPs [150,151]. Chitosan is very attractive as this coating itself repels bacteria. Indeed, results revealed a moderate reduction in MRSA biofilm formation and in the percentage of viable adherent bacteria on the AMP-chitosan-coated surface as compared to chitosan-coated and uncoated metal surfaces. These data indicate that AMP hLf1-11 and Dhvar5 in the correct orientation (and specifically if an optimal spacer is applied) in the coating maintained their antibacterial activity. Another method for coating both metal and organic surfaces with AMPs takes advantage of the adhesive properties of molecules comprising a catechol (DOPA) and amine (e.g., lysine) group to form a spontaneous film on a variety of surfaces, including organic polymers, metals and ceramics [153], using a simple dip coating technique. A couple of studies used polydopamine to coat the surface of silicone catheters [154] and titanium implants [155,156] with AMPs. Lim et al. immobilized the CWR11 peptide onto silicone via a polydopamine coating and found excellent bactericidal and anti-adherence properties of the AMP-coating against *S. aureus* and *P. aeruginosa* [154]. In agreement, CWR11 functionalized catheters displayed excellent antibacterial activities through a period of at least 21 days without hemolytic or cytotoxic effects. Tan et al. prepared a polydopamine coating functionalized with the peptide SESB2V on titanium and determined the bactericidal effect of the immobilized AMP [156]. Results revealed that mean life/dead ratios for *S. aureus* and *P. aeruginosa* on pristane titanium were significantly higher than on AMP-titanium substrates. More importantly, there was a lower incidence and a lesser extent of infection with *S. aureus* and *P. aeruginosa* on rabbit corneas with the AMP-functionalized titanium films than those with unprotected titanium implants. Its efficacy was greater against *S. aureus* than against *P. aeruginosa*. In this keratitis model, the bactericidal effect of immobilized peptide SESB2V was comparable to that of 0.3% gatifloxacin eye drops (every 2 h) and 33 mg of cefazolin combined with 4 mg of gentamicin administered subconjunctively once a day. Others adapted the polydopamine coating technique by using a gelatin-based hydrogel modified with DOPA motifs, further referred to as Gel-DOPA, and functionalized it with HHC-36 [155]. When applied on top of the peptide-containing Gel-DOPA-coated titanium plates, but not the control Gel-DOPA-coated titanium plates, the bacterial counts for *S. aureus*, *S. epidermidis*, *P. aeruginosa* and *E. coli* were reduced by >99%. In addition, the peptide released from Gel-DOPA-coated titanium plates was highly effective in killing these bacteria.

#### 3.4.2. Polymeric Brushes

Polymeric brushes are macromolecular structures with polymeric chains that are from one end chemically coupled to surfaces and from the other end coupled to AMPs. The polymeric brush provides flexibility between the AMP and the surface and increases the density of the AMP on the surface, thereby reducing the impact of surface confinement. A number of studies have utilized the polymeric brush technology to equip titanium surfaces and silicone surfaces with an AMP (Table 4). Gao et al. compared several compositions of copolymer brushes and reported that poly(DMA-*co*-APMA) copolymer brushes were optimal for AMP immobilization [157]. Yu et al. coupled Tet20 and E6 to poly(DMA-*co*-APMA) copolymer brushes attached to polystyrene nanoparticles [158]. These AMP-functionalized coatings were highly effective against *P. aeruginosa* and *S. aureus*; however, the coated peptides were less effective than peptides in solution. In addition, *S. aureus* adherence onto a polymer brush enriched with E6 and coupled to titanium was moderately (10–40%) reduced compared to uncoated titanium. Others developed polymeric brushes composed of AMP-functionalized block copolymer Pluronic F-127 immobilized onto silicone rubber surface by dip-coating [159]. These antiadhesive surfaces repelled colonizing *S. aureus*, *S. epidermidis* and *P. aeruginosa* and killed those bacteria that adhered to the surface. Yu et al. used an antiadhesive brush poly(DMA-*co*-APMA) copolymer coating on polyurethane to immobilize peptide E6 [149]. This surface coating prevented bacterial adhesion in vitro by up to 99% for *P. aeruginosa* and *S. aureus* and *Staphylococcus saprophyticus* and reduced planktonic bacterial growth by up to 70%. Importantly, in a mouse urinary catheter infection model the AMP-coated catheter was highly effective in preventing *P. aeruginosa* infections by reducing bacterial adhesion to the catheter in urine by 4 logs. Mishra et al. immobilized a potent AMP onto a silicone catheter using an allyl glycidyl ether brush and polyethylene glycol-based chemical coupling [160]. The amount of immobilized peptide was about 6.6 µg/cm^2^ and the coated catheter exhibited excellent antimicrobial activity against *E. coli* and *Enterococcus faecalis* and prevented biofilm formation by these bacterial species. These antibacterial properties were maintained over a period of at least 4 days. Furthermore, Monteiro et al. conjugated the peptide Chain201D and a control peptide to tetra(ethylene) glycol-terminated self-assembled monolayers (EG4-SAM) activated by carbonylimidazole on gold surfaces [148]. Chain201D, but not the control peptide, EG4-SAMs killed (by contact) a high percentage of adherent *S. aureus* and *E. coli*. For example, Godoy-Gallardo et al. immobilized hLf1-11 on titanium surfaces by silanization and with polymer brushes prepared by surface-initiated atom transfer radical polymerization [161]. Results revealed a higher decrease in bacterial attachment on surfaces with polymeric brushes as compared to silanization, which the authors attributed to the capacity of the brushes to immobilize more AMPs. Additionally, these modified surfaces did not affect the viability and proliferation of fibroblasts. Acosta et al. coated titanium surfaces through silanization with engineered protein (elastin-like recombinamers; ELR) containing D-GLI13K [162]. They reported that the presence of AMPs on ELR-coatings decreased biofilm formation by 90% and reduced *Streptococcus gordonii* and *Porphymonas gingivalis* viability in the adherent population significantly. Based on their antiadhesive, antibacterial and biocompatible properties polymeric brush-AMPs coatings are interesting candidates for further development as coatings for metal implants and catheters.

#### 3.4.3. Layer-by-Layer Coatings

The layer-by-layer coating strategy creates multilayer AMP reservoirs on surfaces. This technique is very powerful in protecting metal surfaces and catheters from colonization by biofilm-forming bacteria. Moreover, the multilayer composition permits a controlled release of the AMP over a long time span. Several studies have successfully applied this layer-by-layer technique to develop a coating that protects surfaces from bacterial colonization by biofilm-forming bacteria (Table 4). For example, Shukla et al. incorporated ponericin G1 into a hydrolytically degradable, polyelectrolyte multilayer film on a silicone surface by repeated cycles of sequential deposition of poly β-amino ester/polyanion/ponericin G1/polyanion [163]. These multilayered structures may contain up to 150 µg of peptide/cm^2^ and release the peptide over a period of 10 days. Importantly, this peptide coating prevented *S. aureus* from adhering to the surface. Raman et al. produced a multilayered coating consisting of poly-L-glutamic acid and poly-L-lysine incubated with AMP onto the luminal surface of catheters [164]. The AMP was released over a period of 4 months and was shown to kill 80–90% of the planktonic *Candida albicans* and to decrease *C. albicans* biofilm formation by 83% in vitro. Kazimzadeh-Narbat et al. prepared a three-layered coating on titanium surfaces consisting of TiO_2_ nanotubes oriented vertically on the titanium surface using the drop and dry technique, a thin layer of calcium phosphate by electrolytic deposition, and finally a film of 1-palmitoyl-2-oleoyl-sn-glycerol-3-phosphocholine, with each layer being impregnated with the HHC-36 peptide [165]. The coating was highly effective against *S. aureus* and *P. aeruginosa* in the disk diffusion assay. In vitro, this peptide was released from the coating in 5 days after an initial burst release of 27%. In another study, Tet213 was linked to collagen IV and titanium surfaces were coated with multilayers of the AMP-functionalized collagen using a layer-by-layer technique [26]. The peptide was released from the coating at a steady rate over time for at least 28 days and decreased the growth of *S. aureus* and *P. gingivalis* during the entire interval by 40–55%. Importantly, this coating inhibited *S. aureus* biofilm formation considerably. Another highly effective multilayered coating system comprised three layers of PLGA, dipalmitoylphosphatidylcholine, distearoylphosphatidylcholine and cholesterol (PLEX) mixed with 10% OP-145 peptide [25]. They reported that the peptide was released in a controlled fashion from the coating after an initial burst of 55% and displayed antibacterial activity in vitro. More importantly, in a rabbit nail-related infection model 67% of the rabbits with PLEX OP-145-coated nails had culture-negative nails (as opposed to 31% in control nails), while bone and soft tissue were culture-negative in 67% and 80%, respectively. As expected, this coating was more effective than the PLEX coating without peptide and when OP-145 was injected along *S. aureus*-inoculated silicone elastomer implants in mice. In a follow up study using a murine model for biofilm-associated infection, Riool et al. showed that other, closely related, antimicrobial and antibiofilm peptides (SAAPs) incorporated in a five-layered PLEX coating on titanium/aluminum/nobium (TAN) implants significantly reduced the number of culture positive implants and resulted in ≥3.5 and ≥1.5 log lower *S. aureus* counts on the implants and peri-implant tissues [166]. This PLEX coating provided a daily 0.6% release of SAAP-145 and SAAP-276 up to 30 days after an initial burst release of approximately 50%. These peptide coatings were also found to be highly effective against multidrug resistant *S. aureus* in this model; both peptides reduced implant colonization by 2 logs, whereas the SAAP-276 PLEX coating, but not the SAAP-145 PLEX coating, decreased tissue colonization by 1 log. Together, these data indicate that implant colonization by MRSA can be prevented by PLEX coatings releasing SAAPs. It is tempting to state that SAAPs applied in PLEX coatings may be more effective than when administered as peptide solution.

Finally, polymeric AMP coatings provide many advantages over adsorption or direct immobilization of AMP onto metal and organic (e.g., silicone) materials. It should be noted that the efficacy of coatings equipped with AMPs in vitro are often not challenged in biologically relevant conditions, such as urine, in the presence of proteins, cells of the immune system or under dynamic/flow conditions etc. However, several coatings have been tested in animal models (Table 4).

**Table 4:** Antibacterial effects of polymeric AMP coatings.

AMP	Coating and Release	Surface	Antibacterial Activities In Vitro and In Vivo	Refs.
**Thin layer polymeric coatings**				
Dhvar 5 and hLf1-11	Chitosan by spin coating	Titanium, Gold	- Chitosan films decreased bacterial adherence in vitro - Dhvar5 reduced MRSA adherence by 80%/40% compared to Ti/Ch-Ti	[151]
CRW	Polydopamine coating	Silicone	- Coated catheters completely inhibited *E. coli* growth in vitro and reduced *S. aureus*, *E. coli* and *P. aeruginosa* biofilm formation by 92%	[154]
HHC-36	Hydrogel-polydopamine coating; 37% burst release in 24 h, sustained release for 20 days	Silicone catheter	- Reduced *S. aureus*, *S. epidermidis*, *E. coli*, *P. aeruginosa* viability and adherence on Gel-DOPA-AMP and DOPA-AMP hydrogels, respectively **	[155]
SESB2V	Polydopamine coating	Titanium	- Improved scores in *S. aureus* rabbit cornea infection model ** - Improved scores in *P. aeruginosa* rabbit cornea infection model **	[156]
**Polymeric brushes**				
Tet213	Poly(DMA-*co*-APMA) copolymer brush	Titanium	- Reduction of adherent *P. aeruginosa*	[157]
E6 and Tet20	Poly(DMA-*co*-APMA) copolymer brush	Titanium, polystyrene nanoparticles, quartz	- Highly effective reduction of *P. aeruginosa* and *S. aureus* - Potent killing of adherent *P. aeruginosa* and *S. aureus*	[158]
Peptide	Block copolymer Pluronic F-127	Silicone	- Contact killing of *S. epidermidis* and *P. aeruginosa*, but not *S. aureus*, was enhanced **	[159]
Peptide E6	PDMA-*co*-APMA brush	Polyurethane	- Prevented in vitro adherence of *P. aeruginosa*, *S. aureus* and *S. saprophyticus* by 99% ** - Prevented infection in murine UTI model by reducing bacterial adherence to the catheter by 4 logs ** - Reduced bacterial counts in the urine by nearly 3 logs **	[149]
CysLasio-III	Allyl glycidyl ether and PEG coupling	Catheter	- 2-log reduction in *E coli* and *E. faecalis* viability ** - 60% and 40% reduction in *E faecalis* and *E. coli* biofilm formation ** - Reduced hemolytic activity and cytotoxicity	[160]
Chain201D	Tetra(ethylene) glycol-terminated self-assembled monolayers	Gold	- Chain201D-coated surfaces killed up to 79% of the adherent *E. coli* and *S. aureus*, while peptide-coated surface killed 31%	[148]
hLf1-11	Silanization vs brush of DMA-APMA copolymer prepared by SI-ATRP	Titanium	- Reduced adherence of *Streptococcus sanguis*/*Lactobacillus salivarius* by 16%/30% - 70% inhibition of *S. sanguis* and 66% of *L. salivarius* viability - >50% reduction on biofilm formation (both species)	[161]
D-GL13K	Engineered protein polymers (brush)	Titanium	- 90% reduced *S. gordoni* and *P. gingivalis* biofilm formation for 6 days - Increased percentage of dead bacteria **	[162]
**Layer-by-layer coatings**				
Ponericin G1	Sequential deposition of poly beta amino ester/polyanion/ponericin G1/polyanion; release up to 10 days	Silicone catheters	- Complete inhibition of *S. aureus* adherence in vitro	[163]
β-peptide (ACHC-β3hVal-β3hLys)_3_	~700 nm thick multilayer PGA PLL coating; release over 4 months	Catheter	- Killed 99.9% of the planktonic *C. albicans* and reduced *C. albicans* biofilm formation by 83% in vitro **	[164]
HHC-36	3-layered system; AMP in each layer; burst release, then steady release up to 5 days	Titanium	- Highly effective against *S. aureus* and *P. aeruginosa* in vitro	[165]
Tet213	Multilayered peptide-functionalized collagen; AMP released over at least 28 days	Titanium	- Bactericidal activity against planktonic *P. gingivalis* and *S. aureus* in vitro	[26]
OP-145	PLEX; two layers; In 48 h, 55% release, then 1% daily release for 30 days	Titanium-aluminum (7%)-niobium (6%)	- Significantly more culture-negative nails in a rabbit intramedullary nail-related infection model after 28 days - 67% and 80% more culture-negative bone and soft tissue samples	[25]
SAAP-145	PLEX; 5 layers; initial burst >50%, constant release of 0.6% daily up to day 30	Titanium	- >50% reduced number of mouse culture-positive subcutaneous titanium implants - 3-log reduction in *S. aureus* on implants and peri-implant tissue - 3-log reduction in doxycycline-resistant *S. aureus* on implants	[166]
SAAP-276	PLEX; 5 layers; initial burst >50%, constant release of 0.6% daily up to day 30	Titanium	- >50% reduced number of mouse culture-positive subcutaneous titanium implants - 3- and 1.5-log reduction in *S. aureus* on implants and peri-implant tissue - 3- and 1-log reduction in doxycycline-resistant *S. aureus* on implants/peri-implant tissue	[166]

Acronyms: AMP = antimicrobial peptide, BPTCS = 3-bromopropyl trichlorosilane, DMA-APMA = poly(N,N-dimethylacrylamide-*co*-N-(3-aminopropyl)methacrylamide hydrochloride), PEG = polyethylene glycol, PGA PLL = poly-L-glutamic acid poly-L-lysine, PLEX = polymer-lipid encapsulation matrix, SI-ATRP = surface-initiated atom transfer radical polymerization; ** compared to nonloaded coatings or no treatment.

## 4. Discussion

This review aimed to provide an overview of lipid and polymeric AMP delivery systems and coatings developed in the last 5 years and to discuss some of the advantages and limitations of these systems against in vitro and in vivo infections. In the above content, we have critically evaluated the antimicrobial properties of polymeric and lipid-based delivery systems and coatings for AMPs in efficacy studies in vitro as well as in vivo. Here, a perspective for developing such formulations into therapeutics will be provided by discussing important limitations of AMP-based nanoformulations and providing several suggestions and recommendations, which may expedite AMP-based DDSs research to the clinic.

### 4.1. Key Challenges in Bringing AMP-Based Nanoformulations to the Clinic

Despite AMP-based DDSs displaying several advantages compared to their nonformulated counterparts, significant challenges remain to be addressed to produce a viable treatment product for clinical applications against infectious diseases. One of the limitations is a lack of standardized testing in biorelevant conditions, which would allow an effective evaluation and comparison between DDSs. Another important aspect that needs to be addressed for development of these systems into therapeutics is the preparation and evaluation of shelf-stable macroformulations. Both challenges are discussed in more detail below.

#### 4.1.1. Lack of Standardized Tests

Although there are several publications on the preparation and in vitro characterization of DDSs, research is lacking on fully testing the activity of AMP-loaded formulations. One of the most commonly occurring limitation is the improper use of controls—in many cases the peptide-loaded systems are compared only to nonloaded systems or no treatment and not compared to the peptide solution. Such comparisons will often show improved antimicrobial efficacy, but do not indicate if peptide activity was lost upon encapsulation.

To determine the antimicrobial activity and toxicity of the delivery system in a manner that allows comparison between studies, standardized in vitro testing in combination with in vivo evaluation is highly necessary. Currently, antimicrobial testing is mainly performed by broth microdilution MIC testing or zone inhibition tests. These are simple and well-established methods; however, both do not necessarily indicate the killing of bacteria, but rather determine the inhibition of bacterial growth. In addition, the MIC is obtained after a fixed overnight incubation time (usually in a biologically irrelevant medium) and is unable to distinguish between partial or complete inhibition and between bactericidal or bacteriostatic mode of action [167]. Most importantly, both methods use planktonic bacteria, while bacterial infections contain both planktonic as well as biofilm-associated bacteria and persisters, which are more resistant to treatments. Here, an overview of biologically relevant in vitro, ex vivo and in vivo tests is presented for inclusion in a standardized evaluation of AMP-based DDSs.

Many complex in vitro setups have already been developed, such as killing assays on planktonic bacteria as well as immature and mature biofilms. These assays could be further expanded using a relevant microenvironment that mimics the clinical situation to understand the suitability of a delivery system for a given application. This could include incorporation of host cells, for example in 3D collagen-elastin matrix models, immune cells and factors, as well as biological fluids, for example proteolytic enzymes, urine or plasma proteins, as the presence of these components are known to affect the antimicrobial activity of AMPs in vivo [9,168,169,170,171,172,173,174]. Similarly, cytotoxicity evaluation should be performed in more complex in vitro cell models that mimic the clinical infection situation, such as 3D skin models [175,176]. In addition, more complex and biorelevant ex vivo topical wound models have been established, including excision wound models [177], burn-wound models [177,178] and tape-stripped skin and intact skin models [175,177], all using human skin. These models allow a performance evaluation without the ethical concerns associated with in vivo experiments.

Moreover, once sufficient evaluation in vitro and ex vivo has been performed, in vivo experiments are needed to evaluate not only antimicrobial efficacy and safety, but also the pharmacokinetics and pharmacodynamics of the DDS, as well as immunological responses. The choice of animal model should be carefully considered before selection. For topical application, this may be rather simple as a range of wound models on the skin of mouse and pig have been developed [179]. However, if the aim is to treat biofilm infection in cystic fibrosis lungs for example, it may be more difficult to obtain relevant models. Although mice can be genetically modified to show cystic-fibrosis-like symptoms, these models fail to accurately mirror human disease severity, leading researchers to develop the model in larger animals, such as ferrets and guinea pigs [180]. Additionally, the lack of guidelines regarding the dosing of nanoparticulate systems in vivo poses a problem, as no standardized definition for NP dose in biological samples (e.g., blood, urine, inside organs) is available [181,182]. Although some in vivo successes have been reported using lipid and polymeric AMP delivery systems, more work is needed to have a clear understanding of the in vivo behavior of these systems. In a time that is increasingly threatened by antibiotic resistance, it is paramount to make the transition from in vitro and ex vivo models to in vivo studies as fast as possible.

#### 4.1.2. Lack of Shelf-Stable Formulations and Evaluations Thereof

Although both nanoparticle-based systems and coatings have shown impressive activity in vitro and some in vivo, the development of a shelf-stable liquid, gel or solid dosage form is still necessary for the translation of a DDS to the clinic. The shelf stability of AMP-based products is important not only due to the sensitive nature of AMPs, which are prone to degradation and hydrolysis, but also due to interactions that may occur between the AMP and the macroformulation. For example, the incorporation of SAAP-148 in a hypromellose gel carrier has shown to reduce the peptide’s performance, with increasing viscosity resulting in a reduced activity [177]. Dijksteel et al. also evaluated various commercially available wound dressings, including traditional gauze, soaked in the peptide SAAP-148 and found that the composition of the wound dressing substantially reduced the activity of the peptide. This was likely due to binding of the AMP and therefore reducing the concentration of peptide available to interact with bacteria.

Other macroformulations have shown more promising results. Hydrogels prepared by cross-linking the AMP epsilon-poly-l-lysine with catechol showed significantly reduced bacterial burden by more than 4 logs in multidrug-resistant *A. baumannii*-infected burn wounds [183]. Chitosan-based hydrogels and polycarbonate-based hydrogels have also shown promising results as wound dressings for AMPs [184,185]. Additionally, hydrogel wound dressings have been shown to contribute to the debridement of wounds by rehydration of nonviable tissue [186], which is necessary for wound healing, making them a desirable macroformulation for the preparation of wound dressings. Although good results have been observed for peptide solutions in some macroformulations, reports are still lacking on macroformulations of DDSs. These findings emphasize the need for, ideally in vivo, evaluation of the AMP delivery system in a final dosage form, as that may significantly affect the performance.

### 4.2. Clinical Applications of AMP Delivery Systems

**Figure 3:**
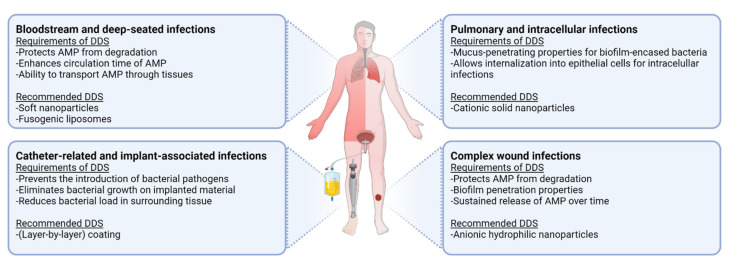
Summary of requirements and recommended DDS for major hard-to-treat infections.

Another important question relates to the choice of AMP-based DDSs for the treatment of different infections. In this section we attempt to identify which lipid or polymeric AMP delivery system or coating is profitable in the fight against and/or prevention of the major hard-to-treat infections. Figure 3 provides an overview of the requirements and recommended DDSs for these infections.

#### 4.2.1. Bloodstream and Deep-Seated Infections

For treatment of infections that are caused by bloodstream-circulating bacteria and deep tissue infections, a DDS that protects the peptide from enzymatic degradation and rapid removal from the circulation and that can be administered systemically is most desirable. Soft nanoparticles, such as nanogels, may be advantageous due to their deformability, which may offer enhanced circulation and aid transport of the delivery system through tissues and to the infection site [187]. Fusogenic liposomes are also advantageous due to their ability to fuse with the bacterial outer membrane and deliver high doses of the AMP directly into the bacteria. However, liposomes are generally quickly opsonized from the bloodstream. A PEG coating on liposomes to make the surface hydrophilic has been shown to increase blood residence time and localization in infected lung tissue [188]. Increased blood circulation has also been reported for PEG-coated PLGA nanoparticles [189]. To further increase blood circulation time, systemically administered nanoparticles should have a diameter larger than 20 nm to avoid filtration by the kidney and smaller than 100 nm to avoid filtration by the spleen and liver [190,191].

#### 4.2.2. Catheter-Related and Implant-Associated Infections

All medical interventions, in particular catheter applications or implantations, are associated with the risk of introducing possible infections. Therefore, there is a need for efficient infection prevention, putting coatings that release AMPs to prevent or treat biomaterial-associated infections in high demand. Coating the implanted material with AMPs may prevent infection occurrence, minimizing post-surgical complications, and the released peptide can eliminate the bacteria already residing in tissues surrounding the implant. Although impressive results have been observed for a range of coatings in vitro, layer-by-layer coatings have shown to be particularly effective for long-term infection prevention, with sustained release reported for up to 4 months for some layer-by-layer coatings [164].

#### 4.2.3. Pulmonary and Intracellular Infections

In the case of specific lung infections, such as chronic bronchitis or cystic fibrosis, a topical administration route by inhalation could be desirable to achieve high local AMP concentrations. Upon inhalation, the AMP has to permeate through the lung mucus and bacterial biofilm to reach the bacteria. In these cases, a mucus-penetrating nanoparticle system could be advantageous, due to the continuous removal of lung mucus by mucociliary clearance. Such a system would be able to diffuse through mucus and deliver high concentrations of AMP in close proximity of the bacterial infection. Additionally, if the aim is treatment of an intracellular infection, such as *M. tuberculosis*, a delivery system that can be internalized in the epithelial cells is desirable. Smaller nanoparticles (>20 nm) induce uptake without requiring endocytic mechanisms [192], though there is a tendency for nanoparticles >100 nm to be more toxic [193]. As a result, larger particles (~200 nm) are usually developed for intracellular delivery. Additionally, solid and cationic particles seem to be preferred, as negatively charged and softer particles show a significantly reduced cellular uptake in a range of cells [187]. Similar results were found for PLGA nanoparticles, where changing the surface charge from negative to more positive significantly improved cytoplasmic delivery [194,195]. The coating of particles, such as liposomes or PLGA nanoparticles with chitosan, a cationic polymer, also significantly increases intracellular delivery [196,197]. Interestingly, quite a few reports on nanogels also indicate good cellular uptake and antimicrobial activity towards intracellular pathogens, despite their soft nature (Table 3).

#### 4.2.4. Complex Wound Infections

Complex wound infections, such as burn wound infections, fracture-related infections and prosthetic joint infections, are associated with biofilms, which protect bacteria from host immune defenses and significantly increase antibiotic resistance. Often, it is unavoidable to treat these wounds by surgical removal of most harmed tissue followed by aggressive antibiotic treatment. For such infections, a classic treatment with cationic antibiotics, such as gentamicin or AMPs, has shown little success due to matrix binding [198]. A DDS that can be administered topically and penetrate through the biofilm, delivering AMPs to the close proximity of bacteria in biofilms for a long period of time would be desirable. Improved penetration into and accumulation in the bacterial biofilm has been observed for negatively charged and hydrophilic particles [28,134], as well as colistin-loaded NLCs [64]. Additionally, a sustained release is also desirable as it reduces the frequency of wound dressing changes and associated pain.

As outlined above, the suitability of a DDS for a given purpose is currently based on reports primarily regarding a single delivery system. Contrasting reports on required properties for some applications only emphasize the need for comparative studies between various DDSs to determine the most desirable properties for this purpose. Back-to-back comparisons of DDSs performed in standardized conditions and on the same models are needed. Such information enables selection of the most suitable DDS for a given application and will substantially increase chances of developing a formulation that can be used in the clinic.

## 5. Future Perspectives

Despite significant progress in the area of AMP drug delivery technology, further work is needed for nanoparticle-based systems or coatings to be developed into clinical therapies. One significant limiting property of many currently described DDSs is a relatively low encapsulation efficiency. This is associated with additional workload due to purification steps that need to be included and a high cost due to significant peptide loss during purification. The encapsulation efficiency can be improved by varying formulation parameters to suit the drug for encapsulation, as has been shown for liposomes [199], PLGA nanoparticles [200] and nanogels [129]. Furthermore, performance parameters of the system, such as the AMP release rate from the nanoparticles, can be controlled by adjusting the composition of the delivery system, such as lipid composition or molecular weight of the polymer used.

Most recently, the trend in the design of AMP delivery systems seems to focus on the design and development of hybrid AMP delivery systems, where the particle surface is functionalized with PEG, biofilm-penetrating ligands, or cell-penetrating peptides to obtain improved targeting and intracellular uptake and simultaneously overcome any possible disadvantages associated with the system itself. Improving existing delivery systems in a precise and targeted way is an excellent approach to improve the targeting of AMPs to their site of action, while building on existing knowledge of currently available delivery systems. Finally, standardized testing and shelf-stable forms of the developed systems are lacking. Therefore, there is an urgent need for clear guidelines on in vitro and ex vivo testing, as well as a need for more relevant in vivo infection models, preferably testing shelf-stable forms of the products, to assess the safety issues and performance of these formulations.

## 6. Conclusions

The increasing development of bacterial resistance to traditional antibiotics has directed research attention to alternative therapies, such as AMPs. However, nonoptimal physicochemical properties, insufficient efficacy and toxicity data, as well as costs, have limited the translation of a large part of AMPs into therapeutic products. Nanoparticle-based AMP therapies and coatings have shown promising results in vitro, aiding delivery of AMPs, not only through bacterial biofilms and in close proximity to bacteria, but also into cells for the treatment of intracellular infections.

The progress in the fields of drug delivery and nanotechnology has led to a vast array of novel nanoparticle systems and coatings, which may allow efficient delivery across biological membranes and an improvement of the antibacterial activity at the site of infection. Nonetheless, the majority of available reports are in vitro-based findings and many challenges still need to be addressed, such as the lack of reproducible infection-specific in vitro and in vivo models, as well as specific guidelines and standards for testing the safety, efficacy and performance of nanoparticle-based therapeutics. Additionally, the translation of these early-stage drug development findings to shelf-stable solid dosage forms is crucial. Overcoming these obstacles will lead to safer and more efficient nanoparticle-mediated AMP therapies entering the clinical phases of drug development.
